# Treatment-Related Adverse Events with PD-1 or PD-L1 Inhibitors: A Systematic Review and Meta-Analysis

**DOI:** 10.3390/life11111277

**Published:** 2021-11-22

**Authors:** Yixi Zhang, Bin La, Baosheng Liang, Yangchun Gu

**Affiliations:** 1 Department of Biostatistics, School of Public Health, Peking University, Beijing 100191, China; zyxlry@pku.edu.cn; 2 School of Public Health, Peking University, Beijing 100191, China; 1710306225@pku.edu.cn; 3 Department of Medical Oncology and Radiation Sickness, Peking University Third Hospital, Beijing 100191, China; pku90201529@126.com

**Keywords:** cancer treatment, PD-1/PD-L1 inhibitors, adverse events, meta-analysis

## Abstract

Objective: to evaluate the risk of treatment-related adverse events of different severity and different system with PD-1 or PD-L1 inhibitors. Methods: randomized controlled trials (RCTs) that using PD-1/PD-L1 for cancer treatment were searched in the PubMed, Embase, Cochrane Library, and Web of Science from 1 January 2019 to 31 May 2021. Adverse events data were extracted from clinical trials website or original article by two authors separately. Meta-analysis was used to determine risk ratio (RR) and 95% confidence interval (95% CI) of adverse events in PD-1/PD-L1 inhibitors groups compared to that of control groups. Subgroup analyses were also performed. Results: a total of 5,807 studies were initially identified and after exclusion, 41 studies were included in meta-analysis. All the trials were international multicenter, randomized, phase II/III clinical trials, with the median follow-up of 27.5 months on average. Analysis of all grade adverse events showed that PD-1/PD-L1 inhibitors treatment significantly increased the risk of immune-related adverse events, including pruritus (RR: 2.34, 95% CI: 1.85–2.96), rash (RR: 1.53, 95% CI: 1.25–1.87), ALT elevation (RR 1.54, 95% CI 1.23–1.92), AST elevation (AST: RR 1.49, 95% CI 1.20–1.85), hepatitis (RR: 3.54, 95% CI: 1.96–6.38) and hypothyroid (RR: 5.29, 95% CI: 4.00–6.99) compared with that of control group. Besides that, PD-1/PD-L1 inhibitors were associated with higher risk of adverse events related to respiratory system including cough (RR: 1.33, 95% CI: 1.21–1.48), dyspnea (RR:1.23, 95% CI: 1.12–1.35) and chest pain (RR: 1.26, 95% CI: 1.07–1.47) compared with that of control groups in our meta-analysis and the dyspnea was taken high risk both in all grade and grade 3 or higher (RR: 1.55, 95% CI: 1.13–2.12). The risk of arthralgia was increased with PD-1/PD-L1 inhibitors (RR: 1.27, 95% CI: 1.10–1.47). Although the risk of myalgia was similar with PD-1/PD-L1 inhibitors and control groups, under subgroup analysis, PD-1/PD-L1 inhibitors decreased the risk of myalgia (RR: 0.56, 95% CI: 0.45–0.70) compared with that of chemotherapy. Conclusions: our results provide clear evidence that the risk of treatment-related adverse events in PD-1 or PD-L1 varies widely in different system. In particular, when using PD-1/PD-L1 inhibitors for oncology treatment, besides the common immune-related adverse events like pruritus, rash, hepatitis, and hypothyroid, the respiratory disorders and musculoskeletal disorders, such as cough, dyspnea, arthralgia, and myalgia, should also be taken into consideration.

## 1. Introduction

In recent years, cancer patients gained increasingly significant benefits from immunotherapy, due to its remarkable clinical efficacy and durable response [1]. Up to now, the FDA approved PD-1 inhibitors nivolumab, pembrolizumab, and PD-L1 inhibitors atezolizumab, avelumab, and durvalumab in clinical trials [2]. In China, a number of PD-1 or PD-L1 inhibitors successfully entered the Medicare list, such as sintilimab, tislelizumab, and camrelizumab for the treatment of metastatic colorectal cancer, nonsmall cell lung cancer, Hodgkin’s lymphoma, and metastatic melanoma.

The PD-1/PD-L1 axis can be modulated by various signals in cancer cells because of higher expression of PD-1/PD-L1 in a large number of solid cancers, which plays a critical role in oncology treatment [3]. In nonsmall-cell lung cancer, immune checkpoint inhibitors show very robust efficacy over docetaxel in overall survival, whether combined with chemotherapy or not [4,5,6]. The combination of PD-1/PD-L1 immune checkpoint inhibitors with first-line chemotherapy provide a significant benefit for extensive-stage small cell lung cancer in overall survival, progression-free survival and objective response rate [7]. For the treatment of advanced renal cell carcinoma and gastroesophageal cancer, PD-1/PD-L1 inhibitors were proved to improve antitumor activity and overall survival [8,9,10].

Although PD-1/PD-L1 drugs outperformed in prolonging patients’ survival, research on drug toxicity and adverse events is limited. With the increase use immune checkpoint inhibitors, there were more and more reports on immune-related adverse events [11]. In a meta-analysis of single-arm PD-1/PD-L1 inhibitors, Wang et al. found that fatigue was the most common all-grade treatment-related adverse events, followed by pruritus and diarrhea [12]. A Cochrane systematic review concluded that the frequency of Grade 3–4 adverse events of single immune checkpoint inhibitor was low, but they did not report specific adverse events [13]. Baxi et al. pointed out that musculoskeletal problems may be a common adverse event with anti-PD-1 drugs, while further investigation was required [14]. Although different PD-1 and PD-L1 inhibitors are accompanied by various treatment-related adverse events, there seems to be no difference between them [12,15].

Treatment-related adverse events refer to an unfavorable change in the health of a participant in clinical trials, which is an important issue in oncology [16]. A comprehensive understanding of adverse events with PD-1 and PD-L1 inhibitors is essential to clinical practice. In this study, we conducted a systematic review and meta-analysis to evaluate the relative risk of treatment-related adverse events with PD-1 and PD-L1 inhibitors in different severity and different system compared with that of control group. We also performed subgroup analysis to investigate the safety of PD-1/PD-L1 inhibitors.

## 2. Methods

### 2.1. Search Strategy

We followed the Preferred Reporting Items for Systematic Reviews and Meta-Analyses (PRISMA). A comprehensive literature search was performed, independently by two authors (Z.Y. and La B.), under four databases, PubMed, Embase, Cochrane Library, and Web of Science, from 1 January 2019 to 31 May 2021. Taking PubMed as an example, the following search terms were used for study retrieval: (“Immune Checkpoint Inhibitors”[mh] OR “immune checkpoint inhibitor”[tiab] OR *checkpoint inhibitor*[tiab] OR checkpoint block* OR “PD 1”[tiab] OR “PD L1”[tiab] OR “anti PD-1”[tiab] OR “anti-PD-L1”[tiab] OR “nivolumab”[mh] OR “nivolumab”[tiab] OR “Opdivo”[tiab] OR “ONO4538”[tiab] OR “MDX-1106”[tiab] OR “BMS-936558”[tiab] OR “pembrolizumab” [Supplementary Concept] OR “SCH-900475”[tiab] OR “keytruda”[tiab] OR “MK-3475”[tiab] OR “lambrolizumab”[tiab] OR “atezolizumab” [Supplementary Concept] OR “atezolizumab”[tiab] OR “MPDL-3280A”[tiab] OR “Tecentriq”[tiab] OR “RG-7446”[tiab] OR “avelumab” [Supplementary Concept] OR “avelumab”[tiab] OR “MSB0010682”[tiab] OR “bavencio”[tiab] OR “MSB0010718C”[tiab] OR “durvalumab”[Supplementary Concept] OR “MEDI4736”[tiab] OR “imfinzi”[tiab]) AND (((clinical trial[pt] OR “clinical trial”[tiab]) OR (clinical trials as topic [mesh: noexp]) OR (randomized [tiab]) OR (randomly [tiab]) OR (trial [ti])) NOT (animals [mh] not humans [mh])). We did not impose any restrictions on type of article, language, design method, cancer type, or number of populations at risk. A similar search strategy and search terms were repeated in Embase, Cochrane Library, and Web of Science, respectively. In addition, reference lists of potentially relevant reports and reviews were screened to identify other eligible studies. We combined the search results in a bibliographic management software (EndNote X9).

### 2.2. Study Selection

Three reviewers (Z.Y., L.B., and G.Y.) independently identified articles eligible for in-depth examination using the following predefined inclusion and exclusion criteria: (1) study design: randomized controlled clinical trials with the purpose of cancer therapy were considered. We excluded case reports, case series, single-arm cohort studies, reviews and meeting abstracts, but we had no restriction on cancer type; (2) type of interventions: participants were treated with a single-agent PD-1 or PD-L1 inhibitor in treatment group; (3) type of outcomes: we focused on treatment-related adverse events, so the included studies should display reported tabulated data on treatment-related adverse events in clinicaltrail.gov or in the full article; (4) published in English. Two authors (Z.Y. and La B.) screened all titles and abstracts for full text reviews. Disagreements were resolved by consensus involving three authors (Z.Y., L.B., and G.Y.).

### 2.3. Data Extraction

Data from each study were extracted by two authors (Z.Y. and La B.). Differences were resolved by consensus. The trial name, phase, cancer type, PD-1 and PD-L1 inhibitor used, dose escalation, dose schedule, number of patients, median follow-up, and number of all treatment-related adverse events were obtained from each included study. Treatment-related adverse events that we care about included general disorders (fatigue, fever, headache), skin disorders (pruritus, rash), respiratory disorders (cough, dyspnea, chest pain, pneumonia), gastrointestinal disorders (loss of appetite, nausea, vomiting, diarrhea, constipation, abdominal pain), liver disorders (ALT elevation, AST elevation, hepatitis), endocrine disorders (hypothyroid), musculoskeletal disorders (myalgia, arthralgia), blood disorders (anemia, neutrophil decrease). Serious adverse events (grade 3 or higher) and other adverse events (grade 1–2) were both extracted.

### 2.4. Outcomes

Our primary outcome was the incidence of different type of treatment-related adverse events. We recorded data on adverse events reported as serious or other on clinical trials website. For data extracted from published reports, we identified grade 3 or higher as serious and grade 1–2 as other. If the study did not report a specific adverse event, we assumed that the event did not occur.

### 2.5. Risk of Bias Assessment

Two authors (Z.Y. and La B.) evaluated the risk of bias with regard to adverse event outcomes by using the tool recommended by the Cochrane Collaboration Handbook. The bias assessment was divided into seven aspects: random sequence generation (selection bias), allocation concealment (selection bias), blinding of participants and personnel (performance bias), blinding of outcome assessment (detection bias), incomplete outcome data (attrition bias), selective reporting (reporting bias), and other bias, which were presented in a graph with high risk (red color), green color (low-risk), and yellow color (unclear).

### 2.6. Data Synthesis and Analysis

For each included study, we calculated risk ratio and 95% confidence interval (95% CI) for incidence rate in the intervention arm compared with that of control, based on the reported number of events and sample size. We used the I^2^ index to examine heterogeneity across trials for each outcome. If significant heterogeneity was not present (*p* > 0.1), pooled risk ratio and 95% CI were estimated with a fixed effect model using the inverse variance method. A random effect model using the inverse variance was used to calculate pooled risk ratio and 95% CI if significant heterogeneity was presents (*p* < 0.1). If a study included more than one intervention arm, we separately compared each intervention arm with the control arm. For example, Roy S. Herbst reported 2 mg/kg and 10 mg/kg for pembrolizumab [17], and Caroline Robert reported q2w and q3w for pembrolizumab [18]. We conducted subgroup analysis by different immune checkpoint inhibitors, different treatment frequency, and different control groups. In subgroup analysis of different control groups, we delivered control groups into chemotherapy, nonchemotherapy, ipilimumab and placebo four subgroups. The trials using “chemotherapy” or “docetaxel” terms as their control were considered in chemotherapy subgroup. “Non-chemotherapy” subgroup included bevacizumab, brentuximab vedotin, dacarbazine, and everolimus as their control groups. We applied the Cochrane Collaboration’s tool to assess the risk of bias. Statistical analyses were conducted using ‘meta’ package in R-4.0.3. The evaluation of bias was based on Review Manager 5.4.

## 3. Results

### 3.1. Results of the Search

**Figure 1:**
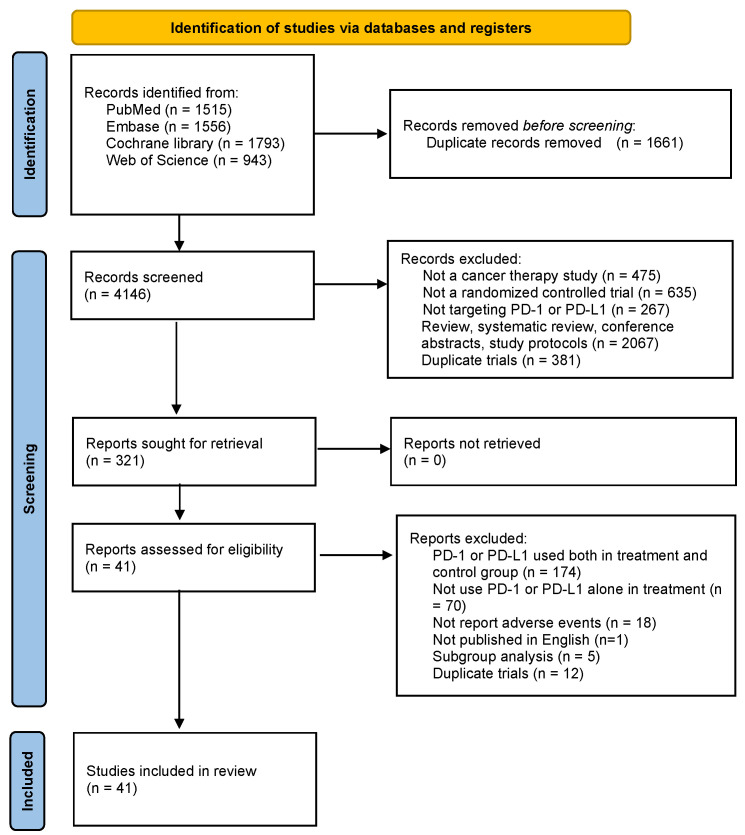
Flow diagram of search and study selection.

Figure 1 shows that the initial research yielded 5,807 relevant articles. After screening and eligibility assessment, we finally included 41 studies in the meta-analysis, a total of 13,232 patients in treatment group and 11,670 in control group. To facilitate the calculation, for the studies with controls of two or more treatments, we divided them into two treatment-control groups [17,18]. Finally, we included 45 clinical trials in the meta-analysis. All trials were international multicenter, randomized, phase II/III clinical trials with the median follow-up of 27.5 months on average. The PD-1 and PD-L1 inhibitors used included nivolumab (*n* = 14), pembrolizumab (*n* = 16), atezolizumab (*n* = 6), avelumab (*n* = 3), durvalumab (*n* = 2), camrelizumab (*n* = 1) and cemiplimab (*n* = 1). The trials involved the treatment of gastrointestinal cancer (*n* = 8), head and neck squamous cell carcinoma (*n* = 4), melanoma (*n* = 6), lung cancer (*n* = 16), urothelial carcinoma (*n* = 5), and other cancer (*n* = 4). Detailed information is summarized in Table 1.

### 3.2. Quality Assessment

**Figure 2:**
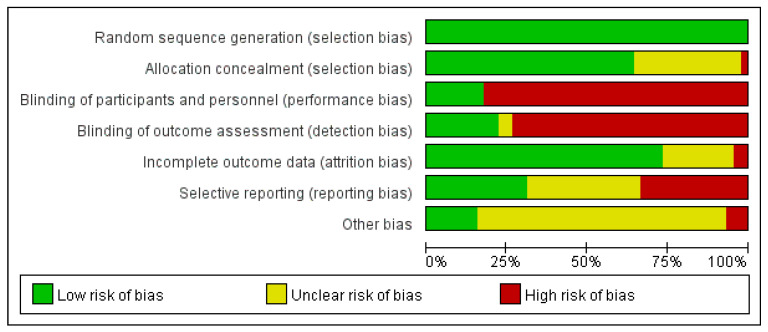
Risk of bias graph: two authors’ judgements about each risk of bias item presented as percentages across all included studies.

Figure 2 shows the risk of bias evaluated by two authors and Figure S1 summaries the bias of every included trial. All RCTs claimed randomization when grouping. High performance bias and detection bias were considered as seven trials explicitly stated double-blind when grouping and the rest were all open-label. We considered high reporting bias because the primary outcome of many RCTs is to evaluate the treatment effect, such as overall survival (OS) or progression-free survival (PFS), rather than adverse events.

### 3.3. Results of Treatment-Related Adverse Event

#### 3.3.1. All Grade Adverse Events

**Figure 3:**
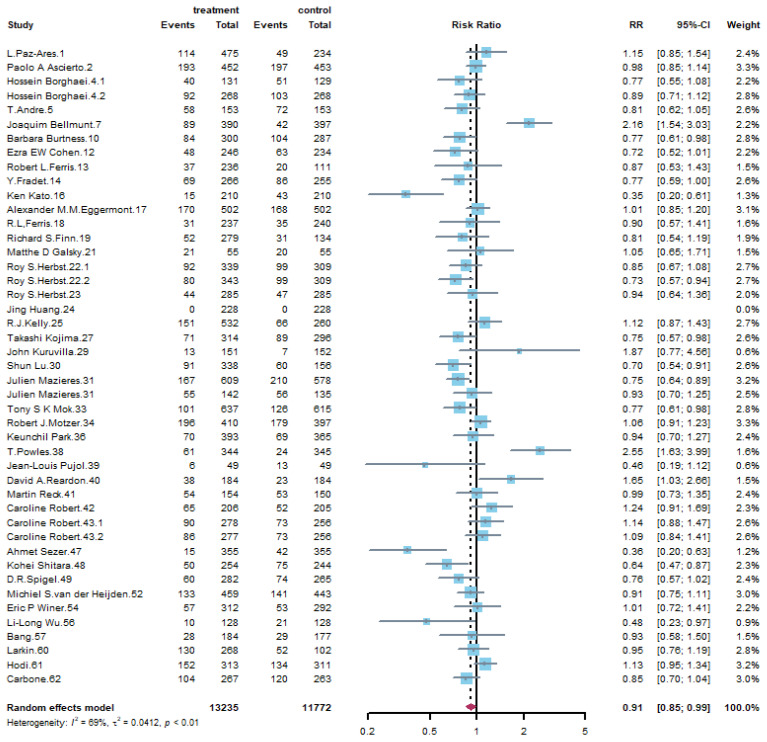
Forest plot of all grade fatigue for PD-1/PD-L1 inhibitors compared with that of control groups.

**Figure 4:**
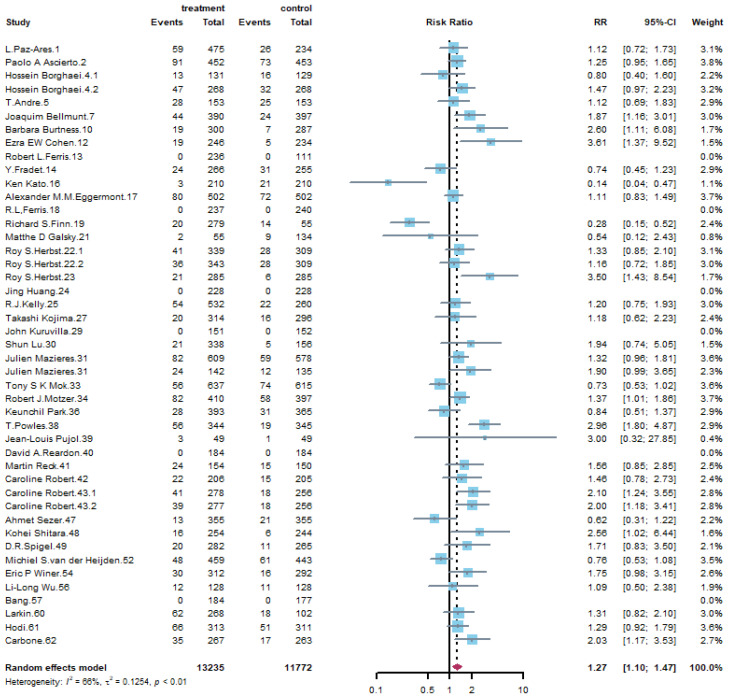
Forest plot of all grade arthralgia for PD-1/PD-L1 inhibitors compared with that of control groups.

Table 2 summarizes the results of all grades’ adverse events with overall risk ratio, 95% confidence intervals (95% CI), and assessment of heterogeneity. For general disorders, fatigue was slightly reduced by PD-1 or PD-L1 inhibitor treatment (RR: 0.91, 95% CI: 0.85–0.99) and Figure 3 demonstrates the corresponding forest plot. It is interesting that PD-1 or PD-L1 inhibitors showed lower risk of peripheral neuropathy compared with control groups (RR: 0.23, 95% CI: 0.16–0.33). For musculoskeletal disorders, arthralgia is another remarkable finding that PD-1 or PD-L1 inhibitors had a higher risk compared with control groups (RR: 1.27, 95% CI: 1.10–1.47) and the forest plot was shown in Figure 4. PD-1 or PD-L1 treatment had a great impact on skin disorders. The risk of pruritus was 2.34 (95% CI: 1.85–2.96) times in treatment compared with that of control group, as is shown in Figure S2, and 1.53 (95% CI: 1.25–1.87) times for rash. Regarding the respiratory disorders, PD-1/PD-L1 inhibitors took a higher risk of respiratory disorders, especially for cough (RR: 1.33, 95% CI: 1.21–1.48), dyspnea (RR: 1.23, 95% CI: 1.12–1.35) and chest pain (RR: 1.26, 95% CI: 1.07–1.47). Although the incidence of pneumonia-related symptoms was higher in PD-1 or PD-L1 treatment group, the difference in pneumonia (RR: 0.96, 95% CI: 0.79–1.18) between two groups was not significant, as is shown in Figure S3. As to gastrointestinal disorders, PD-1 or PD-L1 inhibitors were less harmful to gastrointestinal system. The results performed in Figure S4 confirmed that the incidence of nausea was significantly reduced by PD-1/PD-L1 inhibitors (RR: 0.67, 95% CI: 0.57–0.79). The incidence of vomiting decreased one fifth compared with that of control group (RR: 0.79, 95% CI: 0.68–0.92). For liver disorders, PD-1/PD-L1 inhibitors led to apparent liver system damage. Figures S5 and S6 shows that the risk of ALT elevation (RR: 1.58, 95% CI: 1.26–1.99) and AST elevation (RR: 1.56, 95% CI: 1.22–2.00) were significantly increased. What’s more, PD-1/PD-L1 inhibitors were associated with higher risk of hepatitis (RR: 3.54, 95% CI: 1.96–6.38), as is shown in Figure S7. Finally, as to endocrine disorders, the adverse event of treatment on endocrine is mainly thyroid dysfunction. Figure S8 shows that the hypothyroid of using PD-1 or PD-L1 was 5.29 (95% CI: 4.00–6.99) times compared with that of control group.

#### 3.3.2. Grade 3 or Higher Adverse Event

The incidence of adverse events of grade 3 or higher was low-level both in the treatment group and the control group. Table 3 summarizes the number of trials, corresponding risk ratio and heterogeneity analysis of grade 3 or higher adverse events using PD-1/PD-L1 inhibitors. It is apparent from this table that PD-1/PD-L1 inhibitors took highly risk in serious dyspnea compared with that of control groups (RR: 1.55, 95% CI: 1.13–2.12). No statistically significant difference was found in the incidence of serious general adverse events (fatigue: RR = 0.78, 95% CI: 0.54–1.13; fever: RR = 1.19, 95% CI: 0.91–1.56). The PD-1/PD-L1 outperformed comparators in gastrointestinal disorders and blood disorders but failed to do so with the liver system. In gastrointestinal disorders, the incidence of nausea, vomiting, and diarrhea were associated with better improvement in treatment group than that in control, with the risk ratio and 95% CI of 0.60 (0.39–0.91), 0.56 (0.38–0.83), and 0.57 (0.44–0.74), respectively. With respect to blood disorders, PD-1 or PD-L1 inhibitors greatly reduced the risk of anemia (RR: 0.50, 95% CI: 0.35–0.71) and neutrophil decrease (RR: 0.09, 95% CI: 0.06–0.16). However, using PD-1 or PD-L1 inhibitors showed an increased risk of hepatitis with RR equal to 3.45.

### 3.4. Results of Subgroup Analysis

#### 3.4.1. Subgroup Analysis by Immune Checkpoint Inhibitors 

**Figure 5:**
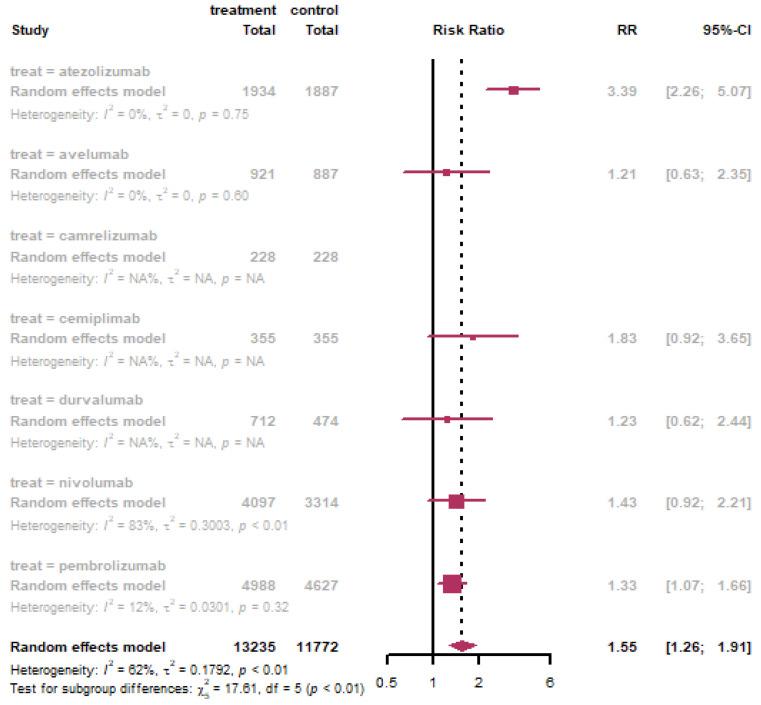
Subgroup analysis of AST elevation in different immune checkpoint inhibitors.

There were 34 trials used PD-1 inhibitors included camrelizumab, cemiplimab, nivolumab, and pembrolizumab, 11 trials for PD-L1 inhibitors with atezolizumab, avelumab, and durvalumab. Subgroup analysis demonstrated PD-L1 inhibitors associated with higher risk of fever (RR: 1.56, 95% CI: 1.24–1.97; see Figure S9) and headache (RR: 1.55, 95% CI: 1.19–2.91; see Figure S10) For PD-L1 inhibitors versus PD-1 inhibitors, the risk of ALT elevation and AST elevation were both higher (RR: 2.10 versus 1.48; 2.34 versus 1.39, respectively). To better estimate the safety of different checkpoint inhibitors, we then conducted subgroup analysis by single drugs. We found that atezolizumab was also associated with the highest risk of AST elevation (RR: 3.39, 95% CI: 2.26–5.07) and there were 5 trials using atezolizumab reported AST elevation (Figure 5).

#### 3.4.2. Subgroup Analysis by Treatment Frequency

According to the frequency of treatment, all the trials were divided into two subgroups, 19 trials every 2 weeks (q2w) and 26 trials every 3 weeks (q3w). Except for myalgia, both groups showed consistent results of treatment-related adverse events. The findings indicated that treatment with q2w led to a significant risk in myalgia (RR: 1.48, 95% CI: 1.03–2.15), while a lower risk of myalgia was favored by q3w (RR: 0.71, 95% CI: 0.53–0.97; Figure S11).

#### 3.4.3. Subgroup Analysis by Control Group

**Figure 6:**
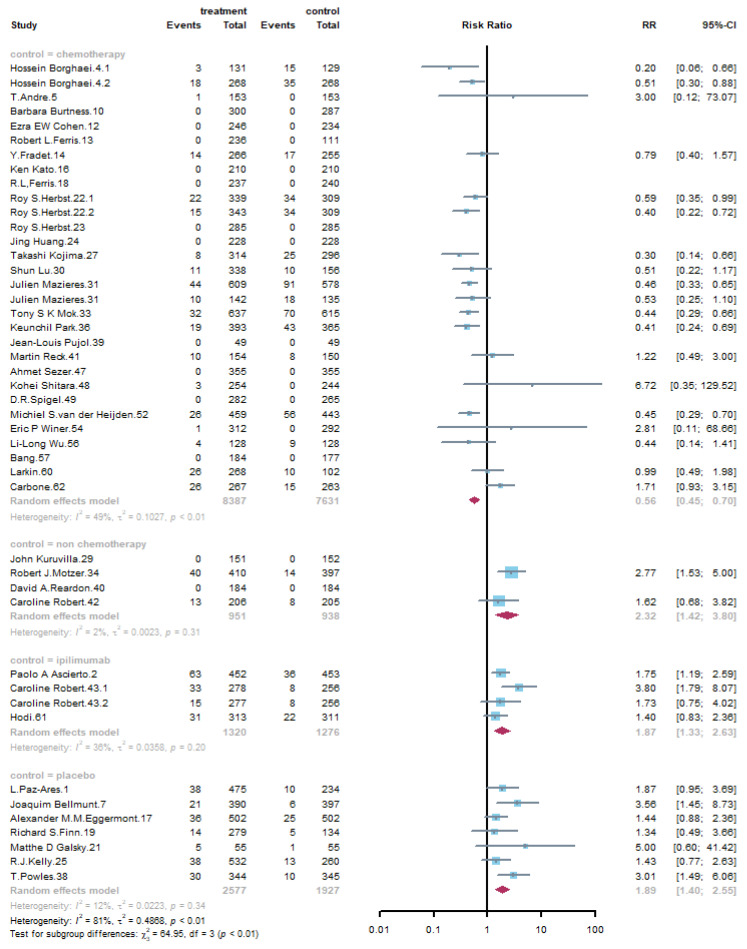
Forest plot of subgroup analysis of myalgia in different control groups.

A total of 30 trials used chemotherapy as control group, and 4 trials entered non-chemotherapy. Another 4 trials used ipilimumab and the rest belonged to placebo. When stratifying trials according to control groups, the result of myalgia is quite different between chemotherapy group and other groups. Compared with that of chemotherapy, PD-1/PD-L1 inhibitors were associated with a lower risk of myalgia (RR: 0.56, 95% CI: 0.45–0.70), but compared with other subgroups, the result was in contrary, as is shown in Figure 6. At the same time, immune-related adverse events were of higher risk when compared with that of chemotherapy groups, such as ALT elevation (RR: 1.56, 95% CI: 1.22–1.99), AST elevation (RR: 1.67, 95% CI: 1.33–2.10), and pruritus (RR:2.83, 95% CI: 2.27–3.51). For other treatment-related adverse events, PD-1/PD-L1 inhibitors increased the risk of cough (RR: 1.33, 95% CI: 1.23–1.44) and dyspnea (RR: 1.26, 95% CI: 1.17–1.37) compared with that of chemotherapy, as is shown in Figures S12 and S13.

## 4. Discussion

In this study, we performed a systematic review and meta-analysis of treatment-related adverse events of PD-1/PD-L1 inhibitors in randomized clinical trials. We found that in all-grade adverse events, PD-1/PD-L1 inhibitors were associated with significant increase in respiratory disorders like cough, dyspnea, and chest pain. What’s more, from our analysis, in addition to focusing on immune-related adverse events, treatment-related adverse events such as arthralgia and myalgia should not be ignored.

We compared the safety of the targeted PD-1/PD-L1 inhibitors with that of the corresponding control group. The results implied that PD-1/PD-L1 inhibitors were associated with a lower risk of all grade or grade 3 or higher treatment-related adverse events. Fatigue is the most common treatment-related adverse event of PD-1 or PD-L1 inhibitors. In single-agent studies, the incidence of fatigue with anti-PD-1 drugs was 18–26% [12]. Normally, fatigue is mild and has nothing to do with other systemic symptoms [16]. Patients treated with PD-1/PD-L1 inhibitors had less likelihood of loss of appetite, nausea, vomiting, and diarrhea, indicating that they may be gastrointestinal-friendly. Meanwhile, immune-related adverse events were frequently reported with PD-1/PD-L1 inhibitors, including rash, pruritus, colitis, aspartate aminotransferase elevation and hypothyroidism [9,58]. Consistent with De Velasco G’s results [59], our study also found that PD-1/PD-L1 inhibitors were associated with more all-grade rash, AST elevation, and hypothyroidism. Skin rash is the most common adverse reaction associated with immune checkpoint therapy. It may be related to Stevens Johnson syndrome and epidermal necrosis, or it may be the blocking effect of drugs on patients’ tumor cells and other common antigens at skin nodes [12]. For patients with severe skin diseases, early dermatological diagnosis and evaluation are recommended. What’s more, a PD-L1 inhibitor plus chemotherapy may decrease the skin reaction compared with a PD-L1 inhibitor alone [60]. For further research, we can discuss the adverse events of combined therapy.

The liver damage is mainly manifested in the undifferentiated increase of AST and ALT, and the pathological appearance of induced hepatitis and ipilimumab are similar [61]. In subgroup analysis, we observed higher risk of AST elevation in PD-L1 inhibitors compared with that of nonimmune checkpoint inhibitors. This outcome is contrary to that of Sonpavde et al. [62] who found a lower risk of ALT elevation for anti-PD-L1 inhibitors versus PD-1 inhibitors. Actually, the difference of two meta-analysis may come from the different methods used in meta-analysis. For the previous study, they used indirect incidence rate and were hypothesis-generating [60,62], while our study used randomized controlled trials, so we could calculate the relative risk.

The most important clinically relevant finding was that PD-1/PD-L1 inhibitors were associated with more respiratory events like all grade cough, all grade and grade 3 or higher dyspnea. Previous single-arm trial CheckMate 063 with anti-PD-1 inhibitors reported 5% patients suffering from dyspnea [63]. Another observational study also noted that 1.4% patients developed cough and 0.8% dyspnea after treatment with nivolumab or pembrolizumab [64]. Our results provided evidence that compared with chemotherapy, PD-1/PD-L1 inhibitors increased the risk of cough and dyspnea.

Moreover, the results of this meta-analysis are notable that arthralgia took a higher risk in PD-1/PD-L1 inhibitors (RR = 1.27). Analysis of KEYNOTE-028 trials found that arthralgia was the most common treatment-related adverse events (19.2%) in pembrolizumab [65]. Baxi et al. discovered higher rates of musculoskeletal problems in clinical trials treated with PD-1/PD-L1 inhibitors, however, they failed to conduct a meta-analysis because of incomplete data [14]. To our knowledge, this is the first meta-analysis focused on musculoskeletal disorders. In our study, there were 39 trials all reporting adverse events related to musculoskeletal system, therefore we could do a meta-analysis and then we checked the risk of myalgia and arthralgia. PD-1/PD-L1 inhibitors were associated higher risk of all grade arthralgia (RR = 1.27), however, the pathogenic mechanism was unclear, which call for more molecular biology research. On the other hand, PD-1/PD-L1 decreased the risk of myalgia (RR = 0.56) compared with that of chemotherapy subgroup, but the risk increased with that of other subgroups. Because myalgia is a common adverse event with docetaxel [66], and thus, in chemotherapy subgroup, the lower risk is acceptable.

Our findings have important implications for clinical oncology treatment choices from the standpoint of patient counseling. From the results of subgroup analysis, we found that the risk of adverse events has a certain difference between PD-1 and PD-L1 inhibitors. If patients choose PD-L1 for cancer treatment, they may face higher risk of fever (RR = 1.56) and headache (RR = 1.55), which may be neglected but equally unbearable compared to that of more serious adverse events. Treatment frequency does not seem to make much difference in the occurrence of adverse events. Our results convinced us that, compared with that of chemotherapy, PD-1/PD-L1 inhibitors reduced the risk of adverse events, especially in digestive disorders and blood system, although they may induce immune-related adverse events. Anyway, when determining a treatment schedule, more consideration can be given to the stage of cancer and patients tolerability, as well as the effectiveness of treatment.

### Limitations

There are some limitations in our meta-analysis. Firstly, the data of adverse events were not taken under uniform standards. The symptoms we analyzed were specialist-reported or patient self-reported, such as fatigue, headache, and nausea. In some cases, there were no report of some adverse events and thus missing data were common. Our analysis method could not deal with missing data. Although we tried out best to collect adverse effect data from https://clinicaltrials.gov/ (18 July 2021) where the trials registered, some of our excerpts were still taken from the literature because no results published in website. Inconsistencies in data sources may led to inaccurate analyses. In addition, we compared different controls together, so the results extension was limited. To solve this question, we conducted subgroup analysis. However, in the subgroup analysis, the number of studies in some subgroups was so small that the results were not credible. Finally, considering the above issues, heterogeneity is a serious and non-negligible problem.

## 5. Conclusions

From our meta-analysis, we found that the risk of treatment-related adverse events in PD-1 or PD-L1 varies widely in different systems. In particular, our results provided evidence that PD-1/PD-L1 inhibitors showed higher risk of respiratory disorders including all-grade cough and chest pain, and all-grade and grade 3 or higher dyspnea. What’s more, compared with that of chemotherapy, PD-1/PD-L1 inhibitors had lower risk of all-grade myalgia but they were related to higher risk of all-grade arthralgia. These treatment-related adverse events should be taken into account when evaluating the safety of immune checkpoint inhibitors.

## Figures and Tables

**Table 1:** Characteristics of included studies.

Study	Author	Year	Disease	Clinical Trial	Phase	Drug	Dose	Frequency	*N*	Data Source (1 = clinical trials website, 2=Article)	Median Follow-Up
1	Carbone et al. [19]	2017	nonsmall cell lung cancer	CheckMate-026	III	nivolumab3	3 mg/kg	q2w	267	1	13.5
2	Bang et al. [20]	2018	gastric or gastro-esophageal junction cancer	JAVELIN gastric 300	III	avelumabmab	10 mg/kg	q2w	184	1	10.6
3	Larkin et al. [21]	2018	melanoma	CheckMate-037	III	nivolumab	3 mg/kg	q2w	268	1	24
4	Hodi et al. [22]	2018	melanoma	CheckMate-067	III	nivolumab	3 mg/kg	q2w	313	1	46.9
5	Barbara Burtness et al. [23]	2019	head and neck squamous cell carcinoma	KeyNote-048	III	pembrolizumab	200 mg	q3w	300	1	11.5
6	Ezra EW. Cohen et al. [24]	2019	head and neck squamous cell carcinoma	KeyNote-040	III	pembrolizumab	200 mg	q3w	246	1	7.5
7	Robert L. Ferris et al. [25]	2019	head and neck squamous cell carcinoma	CheckMate-141	III	nivolumab	3 mg/kg	q2w	236	2	24
8	Y. Fradet et al. [26]	2019	urothelial carcinoma	KeyNote-045	III	pembrolizumab	200 mg	q3w	266	1	27.7
9	Ken Kato et al. [27]	2019	esophageal squamous cell carcinoma	ATTRACTION-3	III	nivolumab	240 mg	q2w	210	2	8
10	Richard S. Finn et al. [28]	2019	hepatocellular	KeyNote-240	III	pembrolizumab	200 mg	q3w	279	1	13.8
11	Tony S K Mok et al. [29]	2019	nonsmall cell lung cancer	KeyNote-042	III	pembrolizumab	200 mg	q3w	637	1	12.8
12	Jean-Louis Pujol et al. [30]	2019	small cell lung cancer	IFCT-1603	II	atezolizumab	1200 mg	q3w	49	2	13.7
13	Martin Reck et al. [31]	2019	nonsmall cell lung cancer	KeyNote-024	III	pembrolizumab	200 mg	q3w	154	1	25.2
14	Caroline Robert et al. [18]	2019	melanoma	KeyNote-006	III	pembrolizumab	10 mg/kg	q2w	278	1	57.7
15	Caroline Robert et al. [18]	2019	melanoma	KeyNote-006	III	pembrolizumab	10 mg/kg	q3w	277	1	57.7
16	L. Paz-Ares et al. [32]	2020	nonsmall cell lung cancer	PACIFIC	III	durvalumab	10 mg/kg	q2w	475	1	33.3
17	Paolo A Ascierto et al. [33]	2020	melanoma	CheckMate-238	III	nivolumab	3 mg/kg	q2w	452	1	51.1
18	Hossein Borghaei et al. [34]	2020	nonsmall cell lung cancer	CheckMate-017	III	nivolumab	3 mg/kg	q3w	131	1	69.4
19	Hossein Borghaei et al. [34]	2020	nonsmall cell lung cancer	CheckMate-057	III	nivolumab	3 mg/kg	q3w	268	1	69.5
20	Thierry. Andre et al. [35]	2020	colorectal cancer	KeyNote-177	III	pembrolizumab	200 mg	q3w	153	2	32.4
21	Alexander M.M. Eggermont et al. [36]	2020	melanoma	KeyNote-054	III	pembrolizumab	200 mg	q3w	502	1	15
22	R.L. Ferris et al. [37]	2020	head and neck squamous cell carcinoma	EAGLE	III	durvalumab	10 mg/kg	q2w	237	1	7.6
23	Matthe D Galsky et al. [38]	2020	urothelial carcinoma	HCRN GU14-182	II	pembrolizumab	200 mg	q3w	55	2	12.9
24	Roy S. Herbst et al. [17]	2020	nonsmall cell lung cancer	KeyNote-010	III	pembrolizumab	2 mg/kg	q3w	339	1	31
25	Roy S. Herbst et al. [17]	2020	nonsmall cell lung cancer	KeyNote-010	III	pembrolizumab	10 mg/kg	q3w	343	1	31
26	Roy S. Herbst et al. [39]	2020	nonsmall cell lung cancer	IMpower110	III	atezolizumab	1200 mg	q3w	285	1	42.6
27	Jing Huang et al. [40]	2020	esophageal squamous cell carcinoma	ESCORT	III	camrelizumab	200 mg	q2w	228	2	8.3
28	Takashi Kojima et al. [41]	2020	esophageal cancer	KeyNote-181	III	pembrolizumab	200 mg	q3w	314	1	7.1
29	Shun Lu et al. [42]	2020	nonsmall cell lung cancer	CheckMate-078	III	nivolumab	3 mg/kg	q2w	338	1	25.9
30	Julien Mazieres et al. [43]	2020	nonsmall cell lung cancer	OAK	III	atezolizumab	1200 mg	q3w	609	1	47.7
31	Julien Mazieres et al. [43]	2020	nonsmall cell lung cancer	POPLAR	II	atezolizumab	1200 mg	q3w	142	1	48.6
32	Robert J.Motzer et al. [44]	2020	renal cell carcinoma	CheckMate-025	III	nivolumab	3 mg/kg	q2w	410	1	72
33	T.Powles et al. [45]	2020	urothelial carcinoma	JAVELIN Bladder 100	III	avelumab	10 mg/kg	q2w	344	1	19
34	David A.Reardon et al. [46]	2020	glioblastoma	CheckMate-143	III	nivolumab	3 mg/kg	q2w	184	2	9.4
35	Caroline Robert et al. [47]	2020	melanoma	CheckMate-066	III	nivolumab	3 mg/kg	q2w	206	1	32
36	Kohei Shitara et al. [48]	2020	gastric cancer	KeyNote-062	III	pembrolizumab	200 mg	q3w	254	1	29.4
37	Li-Long Wu et al. [49]	2020	nonsmall cell lung cancer	KeyNote-042 China Study	III	pembrolizumab	200 mg	q3w	128	1	12.8
38	Joaquim Bellmunt et al. [50]	2021	urothelial carcinoma	IMvigor010	III	atezolizumab	1200 mg	q3w	390	1	21.9
39	R.J.Kelly et al. [51]	2021	esophageal or gastroesophageal junction cancer	CheckMate-577	III	nivolumab	240 mg	q2w	532	1	24.4
40	John Kuruvilla et al. [52]	2021	Hodgkin’s lymphoma	KeyNote-204	III	pembrolizumab	200 mg	q3w	151	2	25.7
41	Keunchil Park et al. [53]	2021	nonsmall cell lung cancer	JAVELIN Lung 200	III	avelumab	10 mg/kg	q2w	393	1	35.4
42	Ahmet Sezer et al. [54]	2021	nonsmall cell lung cancer	EMPOWER Lung 1	III	cemiplimab	350 mg	q3w	355	2	10.8
43	D.R.Spigel et al. [55]	2021	small cell lung cancer	CheckMate-131	III	nivolumab	240 mg	q2w	282	1	7
44	Michiel S.van der Heijden et al. [56]	2021	urothelial carcinoma	IMvigor211	III	atezolizumab	1200 mg	q3w	459	1	17.8
45	Eric P Winer et al. [57]	2021	triple negative breast cancer	KeyNote-119	III	pembrolizumab	200 mg	q3w	312	1	31.4

**Table 2:** Results of all grades’ adverse events.

Adverse Events	No. of Trials	RR	95% CI	Test of Heterogeneity	
				*p*	I^2^%
General disorders					
Fatigue	43	0.91	0.85–0.99	<0.01	69
Fever	39	1.12	0.97–1.29	<0. 01	72
headache	37	1.17	1.04–1.32	<0. 01	45
Peripheral neuropathy	32	0.23	0.16–0.33	<0.01	60
Skin disorders					
Pruritus	40	2.34	1.85–2.96	<0.01	87
Rash	42	1.53	1.25–1.87	<0.01	84
Respiratory disorders					
Cough	39	1.33	1.21–1.48	<0.01	61
Dyspnea	39	1.23	1.12–1.35	<0.01	42
Chest pain	35	1.26	1.07–1.47	<0.01	61
pneumonia	40	0.96	0.79–1.18	<0.01	48
Gastrointestinal disorders					
Loss of appetite	39	0.90	0.78–1.04	<0.01	82
nausea	45	0.67	0.57–0.79	<0.01	90
vomiting	41	0.79	0.68–0.92	<0.01	78
diarrhea	45	0.86	0.74–0.99	<0.01	87
constipation	41	0.91	0.81–1.01	<0.01	70
Abdominal pain	32	0.95	0.86–1.05	0.30	10
Liver disorders					
ALT elevation	34	1.58	1.26–1.99	<0.01	70
AST elevation	28	1.56	1.22–2.00	<0. 01	71
Hepatitis	26	3.54	1.96–6.38	1.00	0
Endocrine disorders					
hypothyroid	37	5.29	4.00–6.99	<0.01	59
Musculoskeletal disorders					
myalgia	29	1.00	0.75–1.32	<0.01	81
arthralgia	39	1.27	1.10–1.47	<0.01	66
Blood disorders					
anemia	43	0.58	0.49–0.68	<0.01	87
Neutrophil decrease	32	0.08	0.07–0.10	0.39	5

**Table 3:** Results of Grade 3 or higher adverse events.

Disease	No. of Trials	RR	95% CI	Test of Heterogeneity		
				Q	*p*	I^2^%
General disorders						
fatigue	37	0.78	0.54–1.13	27.04	0.86	0
fever	40	1.19	0.91–1.56	36.34	0.59	0
Respiratory disorders						
dyspnea	35	1.55	1.13–2.12	28.60	0.73	0
pneumonia	39	0.94	0.81–1.09	41.13	0.34	8
Gastrointestinal disorders						
nausea	34	0.60	0.39–0.91	23.53	0.89	0
vomiting	37	0.56	0.38–0.83	30.88	0.71	0
diarrhea	42	0.57	0.44–0.74	62.04	0.02	34
constipation	30	0.66	0.41–1.07	25.61	0.65	0
abdominal pain	36	0.75	0.53–1.05	18.74	0.99	0
Liver disorders						
ALT elevation	25	1.63	0.96–2.77	15.28	0.91	0
hepatitis	26	3.45	1.91–6.23	4.31	1.00	0
Kidney disorders						
kidney injury	31	1.14	0.79–1.62	24.94	0.73	0
Blood disorders						
anemia	40	0.50	0.35–0.71	67.26	0.003	42
neutrophil decrease	22	0.09	0.06–0.16	12.56	0.92	0

## Data Availability

Not applicable.

## Supplementary Materials

The following are available online at https://www.mdpi.com/article/10.3390/life11111277/s1, Figure S1: The risk of bias summary, Figure S2–S13: Forest plots for different adverse events, respectively.
