# Supplementary material for: Treatment-Related Adverse Events with PD-1 or PD-L1 Inhibitors: A Systematic Review and Meta-Analysis
Source: Life (Basel). 2021 Nov 22;11(11):1277. doi: 10.3390/life11111277 (PMC8618590; doi:10.3390/life11111277)
Supplement: Supplementary file 1 [file life-11-01277-s001.zip › Proofreading -- Supplimentary Files/Supplementary figures-proofreading-11.20.pdf]

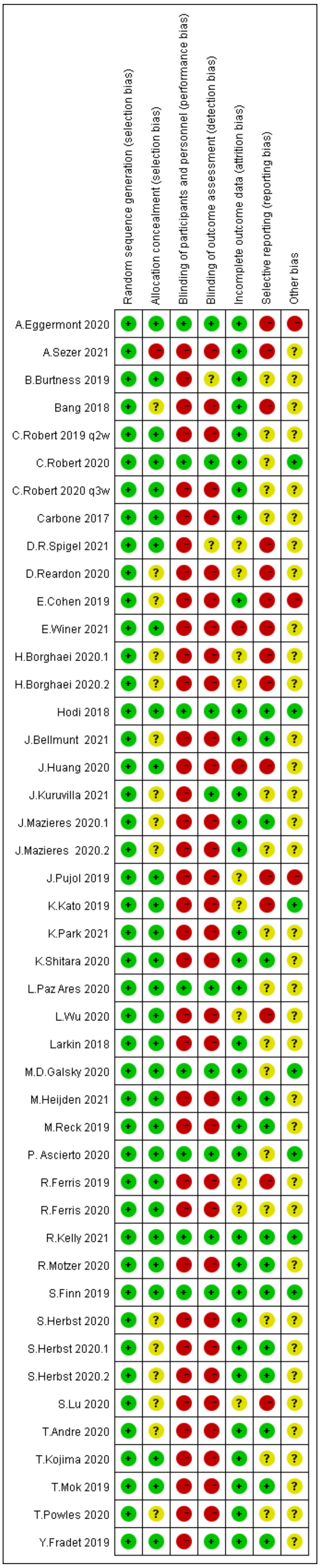

Figure S1. The risk of bias summary

Study	treatment Events	treatment Total	control Events	control Total	RR	95%-CI	Weight
L.Paz-Ares.1	59	475	12	234	2.42	[1.33; 4.42]	2.7%
Paolo A Ascierto.2	137	452	179	453	0.77	[0.64; 0.92]	3.2%
Hossein Borghaei.4.1	10	131	3	129	3.28	[0.92; 11.65]	1.7%
Hossein Borghaei.4.2	33	268	5	268	6.60	[2.62; 16.65]	2.1%
T.Andre.5	25	153	12	153	2.08	[1.09; 3.99]	2.6%
Joaquim Bellmunt.7	92	390	10	397	9.37	[4.95; 17.72]	2.6%
Barbara Burtness.10	33	300	30	287	1.05	[0.66; 1.68]	2.9%
Ezra EW Cohen.12	18	246	18	234	0.95	[0.51; 1.78]	2.6%
Robert L.Ferris.13	19	236	0	111	18.39	[1.12; 301.78]	0.6%
Y.Fradet.14	62	266	14	255	4.25	[2.44; 7.39]	2.7%
Ken Kato.16	0	210	0	210			0.0%
Alexander M.M.Eggermont.17	99	502	58	502	1.71	[1.27; 2.30]	3.1%
R.L,Ferris.18	10	237	9	240	1.13	[0.47; 2.72]	2.2%
Richard S.Finn.19	51	279	16	134	1.53	[0.91; 2.58]	2.8%
Matthe D Galsky.21	13	55	7	55	1.86	[0.80; 4.30]	2.3%
Roy S.Herbst.22.1	32	339	10	309	2.92	[1.46; 5.83]	2.5%
Roy S.Herbst.22.2	41	343	10	309	3.69	[1.88; 7.25]	2.5%
Roy S.Herbst.23	24	285	4	285	6.00	[2.11; 17.07]	2.0%
Jing Huang.24	0	228	0	228			0.0%
R.J.Kelly.25	72	532	16	260	2.20	[1.31; 3.70]	2.8%
Takashi Kojima.27	23	314	8	296	2.71	[1.23; 5.96]	2.4%
John Kuruvilla.29	16	151	8	152	2.01	[0.89; 4.56]	2.3%
Shun Lu.30	32	338	5	156	2.95	[1.17; 7.44]	2.2%
Julien Mazieres.31	58	609	18	578	3.06	[1.82; 5.13]	2.8%
Julien Mazieres.31	16	142	6	135	2.54	[1.02; 6.29]	2.2%
Tony S K Mok.33	62	637	18	615	3.33	[1.99; 5.55]	2.8%
Robert J.Motzer.34	75	410	50	397	1.45	[1.04; 2.02]	3.0%
Keunchil Park.36	26	393	13	365	1.86	[0.97; 3.56]	2.6%
T.Powles.38	59	344	6	345	9.86	[4.32; 22.54]	2.3%
Jean-Louis Pujol.39	3	49	2	49	1.50	[0.26; 8.59]	1.2%
David A.Reardon.40	11	184	9	184	1.22	[0.52; 2.88]	2.3%
Martin Reck.41	23	154	5	150	4.48	[1.75; 11.48]	2.1%
Caroline Robert.42	46	206	24	205	1.91	[1.21; 3.00]	2.9%
Caroline Robert.43.1	68	278	65	256	0.96	[0.72; 1.29]	3.1%
Caroline Robert.43.2	59	277	65	256	0.84	[0.62; 1.14]	3.1%
Ahmet Sezer.47	0	355	0	355			0.0%
Kohei Shitara.48	22	254	8	244	2.64	[1.20; 5.82]	2.4%
D.R.Spigel.49	24	282	4	265	5.64	[1.98; 16.03]	2.0%
Michiel S.van der Heijden.52	64	459	19	443	3.25	[1.98; 5.33]	2.8%
Eric P Winer.54	34	312	12	292	2.65	[1.40; 5.02]	2.6%
Li-Long Wu.56	17	128	3	128	5.67	[1.70; 18.86]	1.7%
Bang.57	0	184	0	177			0.0%
Larkin.60	70	268	3	102	8.88	[2.86; 27.57]	1.8%
Hodi.61	83	313	125	311	0.66	[0.52; 0.83]	3.1%
Carbone.62	34	267	13	263	2.58	[1.39; 4.77]	2.6%
**Random effects model**		**13235**		**11772**	**2.34**	**[1.85; 2.96]**	**100.0%**

Heterogeneity: $I^2 = 87\%$, $\tau^2 = 0.4405$, $p < 0.01$

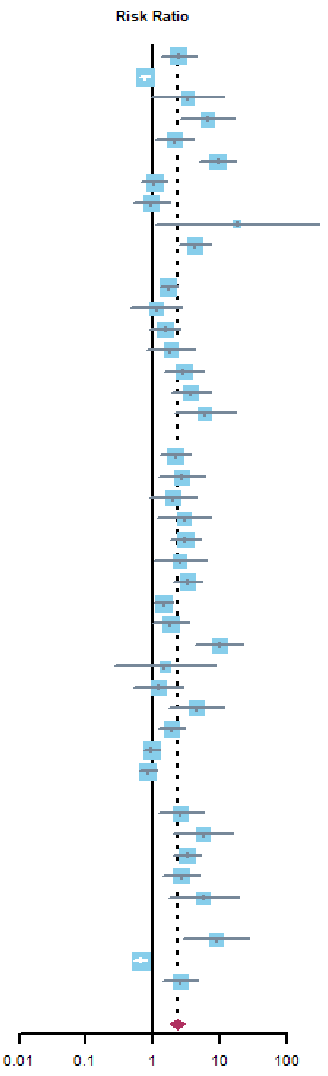

Figure S2. Forest plot of all grade pruritus for PD-1/PD-L1 inhibitors compared with control groups.

Study	treatment Events	treatment Total	control Events	control Total	RR	95%-CI	Weight
L.Paz-Ares.1	71	475	23	234	1.52	[0.98; 2.37]	5.2%
Paolo A Ascierto.2	4	452	6	453	0.67	[0.19; 2.35]	1.9%
Hossein Borghaei.4.1	7	131	10	129	0.69	[0.27; 1.76]	2.8%
Hossein Borghaei.4.2	12	268	13	268	0.92	[0.43; 1.99]	3.5%
T.Andre.5	6	153	1	153	6.00	[0.73; 49.25]	0.8%
Joaquim Bellmunt.7	4	390	2	397	2.04	[0.38; 11.05]	1.2%
Barbara Burtness.10	31	300	30	287	0.99	[0.61; 1.59]	5.0%
Ezra EW Cohen.12	20	246	16	234	1.19	[0.63; 2.24]	4.1%
Robert L.Ferris.13	0	236	0	111			0.0%
Y.Fradet.14	9	266	8	255	1.08	[0.42; 2.75]	2.8%
Ken Kato.16	0	210	0	210			0.0%
Alexander M.M.Eggermont.17	1	502	0	502	3.00	[0.12; 73.47]	0.4%
R.L,Ferris.18	8	237	9	240	0.90	[0.35; 2.29]	2.8%
Richard S.Finn.19	4	279	2	134	0.96	[0.18; 5.18]	1.2%
Matthe D Galsky.21	0	55	0	55			0.0%
Roy S.Herbst.22.1	15	339	16	309	0.85	[0.43; 1.70]	3.8%
Roy S.Herbst.22.2	16	343	16	309	0.90	[0.46; 1.77]	3.9%
Roy S.Herbst.23	14	285	14	285	1.00	[0.49; 2.06]	3.7%
Jing Huang.24	0	228	0	228			0.0%
R.J.Kelly.25	52	532	16	260	1.59	[0.93; 2.73]	4.6%
Takashi Kojima.27	14	314	20	296	0.66	[0.34; 1.28]	3.9%
John Kuruvilla.29	12	151	1	152	12.08	[1.59; 91.75]	0.9%
Shun Lu.30	14	338	5	156	1.29	[0.47; 3.52]	2.6%
Julien Mazieres.31	20	609	34	578	0.56	[0.33; 0.96]	4.6%
Julien Mazieres.31	10	142	3	135	3.17	[0.89; 11.27]	1.9%
Tony S K Mok.33	80	637	56	615	1.38	[1.00; 1.91]	5.8%
Robert J.Motzer.34	28	410	70	397	0.39	[0.26; 0.59]	5.3%
Keunchil Park.36	9	393	19	365	0.44	[0.20; 0.96]	3.4%
T.Powles.38	1	344	0	345	3.01	[0.12; 73.60]	0.4%
Jean-Louis Pujol.39	0	49	0	49			0.0%
David A.Reardon.40	4	184	0	184	9.00	[0.49; 165.97]	0.5%
Martin Reck.41	3	154	9	150	0.32	[0.09; 1.18]	1.8%
Caroline Robert.42	1	206	0	205	2.99	[0.12; 72.86]	0.4%
Caroline Robert.43.1	2	278	1	256	1.84	[0.17; 20.19]	0.7%
Caroline Robert.43.2	1	277	1	256	0.92	[0.06; 14.70]	0.5%
Ahmet Sezer.47	11	355	1	355	11.00	[1.43; 84.75]	0.9%
Kohei Shitara.48	4	254	8	244	0.48	[0.15; 1.57]	2.1%
D.R.Spigel.49	19	282	19	265	0.94	[0.51; 1.74]	4.2%
Michiel S.van der Heijden.52	4	459	7	443	0.55	[0.16; 1.87]	2.0%
Eric P Winer.54	5	312	7	292	0.67	[0.21; 2.08]	2.2%
Li-Long Wu.56	8	128	10	128	0.80	[0.33; 1.96]	2.9%
Bang.57	5	184	2	177	2.40	[0.47; 12.23]	1.3%
Larkin.60	7	268	0	102	5.73	[0.33; 99.36]	0.5%
Hodi.61	0	313	2	311	0.20	[0.01; 4.12]	0.4%
Carbone.62	10	267	17	263	0.58	[0.27; 1.24]	3.5%
**Random effects model**		**13235**		**11772**	**0.96**	**[0.79; 1.18]**	**100.0%**

Heterogeneity: $I^2 = 48\%$, $\tau^2 = 0.1576$, $p < 0.01$

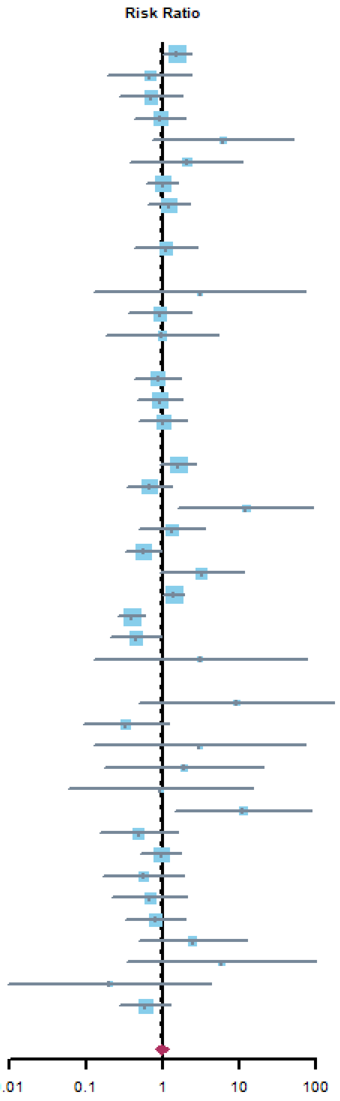

Figure S3. Forest plot of all grade pneumonia for PD-1/PD-L1 inhibitors compared with control groups.

Study	treatment Events	treatment Total	control Events	control Total	RR	95%-CI	Weight
L.Paz-Ares.1	69	475	31	234	1.10	[0.74; 1.63]	2.3%
Paolo A Ascierto.2	111	452	138	453	0.81	[0.65; 1.00]	2.6%
Hossein Borghaei.4.1	20	131	33	129	0.60	[0.36; 0.98]	2.1%
Hossein Borghaei.4.2	67	268	80	268	0.84	[0.63; 1.10]	2.5%
T.Andre.5	23	153	85	153	0.27	[0.18; 0.40]	2.3%
Joaquim Bellmunt.7	52	390	20	397	2.65	[1.61; 4.35]	2.1%
Barbara Burtness.10	50	300	154	287	0.31	[0.24; 0.41]	2.5%
Ezra EW Cohen.12	34	246	45	234	0.72	[0.48; 1.08]	2.3%
Robert L.Ferris.13	22	236	23	111	0.45	[0.26; 0.77]	2.1%
Y.Fradet.14	56	266	73	255	0.74	[0.54; 1.00]	2.5%
Ken Kato.16	4	210	34	210	0.12	[0.04; 0.33]	1.3%
Alexander M.M.Eggermont.17	89	502	73	502	1.22	[0.92; 1.62]	2.5%
R.L,Ferris.18	27	237	41	240	0.67	[0.42; 1.05]	2.2%
Richard S.Finn.19	32	279	20	134	0.77	[0.46; 1.29]	2.1%
Matthe D Galsky.21	9	55	11	55	0.82	[0.37; 1.82]	1.6%
Roy S.Herbst.22.1	75	339	57	309	1.20	[0.88; 1.63]	2.4%
Roy S.Herbst.22.2	65	343	57	309	1.03	[0.75; 1.42]	2.4%
Roy S.Herbst.23	42	285	89	285	0.47	[0.34; 0.66]	2.4%
Jing Huang.24	4	228	81	228	0.05	[0.02; 0.13]	1.4%
R.J.Kelly.25	130	532	62	260	1.02	[0.79; 1.33]	2.5%
Takashi Kojima.27	60	314	85	296	0.67	[0.50; 0.89]	2.5%
John Kuruvilla.29	6	151	20	152	0.30	[0.12; 0.73]	1.5%
Shun Lu.30	26	338	14	156	0.86	[0.46; 1.60]	1.9%
Julien Mazieres.31	112	609	132	578	0.81	[0.64; 1.01]	2.5%
Julien Mazieres.31	33	142	45	135	0.70	[0.48; 1.02]	2.3%
Tony S K Mok.33	74	637	197	615	0.36	[0.28; 0.46]	2.5%
Robert J.Motzer.34	117	410	114	397	0.99	[0.80; 1.24]	2.6%
Keunchil Park.36	56	393	59	365	0.88	[0.63; 1.23]	2.4%
T.Powles.38	54	344	22	345	2.46	[1.53; 3.95]	2.2%
Jean-Louis Pujol.39	5	49	11	49	0.45	[0.17; 1.21]	1.4%
David A.Reardon.40	7	184	9	184	0.78	[0.30; 2.04]	1.4%
Martin Reck.41	20	154	70	150	0.28	[0.18; 0.43]	2.2%
Caroline Robert.42	48	206	97	205	0.49	[0.37; 0.66]	2.5%
Caroline Robert.43.1	73	278	56	256	1.20	[0.89; 1.63]	2.4%
Caroline Robert.43.2	70	277	56	256	1.16	[0.85; 1.57]	2.4%
Ahmet Sezer.47	14	355	91	355	0.15	[0.09; 0.26]	2.1%
Kohei Shitara.48	49	254	134	244	0.35	[0.27; 0.46]	2.5%
D.R.Spigel.49	42	282	56	265	0.70	[0.49; 1.01]	2.4%
Michiel S.van der Heijden.52	106	459	141	443	0.73	[0.58; 0.90]	2.6%
Eric P Winer.54	47	312	87	292	0.51	[0.37; 0.69]	2.4%
Li-Long Wu.56	10	128	24	128	0.42	[0.21; 0.84]	1.8%
Bang.57	35	184	63	177	0.53	[0.37; 0.76]	2.4%
Larkin.60	87	268	43	102	0.77	[0.58; 1.02]	2.5%
Hodi.61	97	313	9	311	10.71	[5.51; 20.82]	1.9%
Carbone.62	80	267	137	263	0.58	[0.46; 0.71]	2.6%
**Random effects model**		**13235**		**11772**	**0.67**	**[0.57; 0.79]**	**100.0%**

Heterogeneity: $I^2 = 90\%$, $\tau^2 = 0.2626$, $p < 0.01$

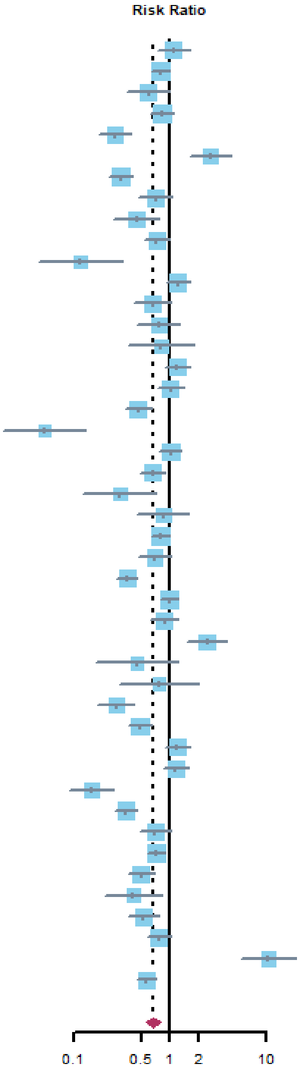

Figure S4. Forest plot of all grade nausea for PD-1/PD-L1 inhibitors compared with control groups.

Study	treatment Events	treatment Total	control Events	control Total	RR	95%-CI	Weight
L.Paz-Ares.1	0	475	0	234			0.0%
Paolo A Ascierto.2	40	452	93	453	0.43	[0.30; 0.61]	4.1%
Hossein Borghaei.4.1	0	131	0	129			0.0%
Hossein Borghaei.4.2	16	268	5	268	3.20	[1.19; 8.61]	2.5%
T.Andre.5	22	153	16	153	1.37	[0.75; 2.51]	3.5%
Joaquim Bellmunt.7	24	390	7	397	3.49	[1.52; 8.01]	2.9%
Barbara Burtness.10	13	300	23	287	0.54	[0.28; 1.05]	3.3%
Ezra EW Cohen.12	0	246	0	234			0.0%
Robert L.Ferris.13	0	236	0	111			0.0%
Y.Fradet.14	1	266	0	255	2.88	[0.12; 70.28]	0.5%
Ken Kato.16	0	210	0	210			0.0%
Alexander M.M.Eggermont.17	37	502	24	502	1.54	[0.94; 2.54]	3.7%
R.L,Ferris.18	11	237	17	240	0.66	[0.31; 1.37]	3.1%
Richard S.Finn.19	50	279	13	55	0.76	[0.44; 1.30]	3.6%
Matthe D Galsky.21	10	55	1	134	24.36	[3.19; 185.79]	1.0%
Roy S.Herbst.22.1	24	339	4	309	5.47	[1.92; 15.59]	2.3%
Roy S.Herbst.22.2	16	343	4	309	3.60	[1.22; 10.66]	2.3%
Roy S.Herbst.23	31	285	16	285	1.94	[1.08; 3.46]	3.5%
Jing Huang.24	0	228	0	228			0.0%
R.J.Kelly.25	43	532	8	260	2.63	[1.25; 5.51]	3.1%
Takashi Kojima.27	22	314	9	296	2.30	[1.08; 4.92]	3.0%
John Kuruvilla.29	5	151	6	152	0.84	[0.26; 2.69]	2.1%
Shun Lu.30	55	338	17	156	1.49	[0.90; 2.49]	3.7%
Julien Mazieres.31	37	609	14	578	2.51	[1.37; 4.59]	3.5%
Julien Mazieres.31	9	142	0	135	18.07	[1.06; 307.40]	0.6%
Tony S K Mok.33	0	637	0	615			0.0%
Robert J.Motzer.34	28	410	23	397	1.18	[0.69; 2.01]	3.6%
Keunchil Park.36	1	393	0	365	2.79	[0.11; 68.19]	0.5%
T.Powles.38	18	344	2	345	9.03	[2.11; 38.60]	1.6%
Jean-Louis Pujol.39	0	49	0	49			0.0%
David A.Reardon.40	10	184	0	184	21.00	[1.24; 355.74]	0.6%
Martin Reck.41	18	154	11	150	1.59	[0.78; 3.26]	3.2%
Caroline Robert.42	11	206	6	205	1.82	[0.69; 4.84]	2.5%
Caroline Robert.43.1	22	278	14	256	1.45	[0.76; 2.77]	3.3%
Caroline Robert.43.2	17	277	14	256	1.12	[0.56; 2.23]	3.2%
Ahmet Sezer.47	18	355	12	355	1.50	[0.73; 3.07]	3.2%
Kohei Shitara.48	13	254	9	244	1.39	[0.60; 3.19]	2.9%
D.R.Spigel.49	18	282	9	265	1.88	[0.86; 4.11]	3.0%
Michiel S.van der Heijden.52	2	459	0	443	4.83	[0.23; 100.24]	0.5%
Eric P Winer.54	23	312	23	292	0.94	[0.54; 1.63]	3.6%
Li-Long Wu.56	27	128	31	128	0.87	[0.55; 1.37]	3.9%
Bang.57	12	184	14	177	0.82	[0.39; 1.73]	3.1%
Larkin.60	26	268	3	102	3.30	[1.02; 10.66]	2.1%
Hodi.61	24	313	16	311	1.49	[0.81; 2.75]	3.4%
Carbone.62	30	267	18	263	1.64	[0.94; 2.87]	3.6%
**Random effects model**		**13235**		**11772**	**1.58**	**[1.26; 1.99]**	**100.0%**

Heterogeneity: $I^2 = 70\%$, $\tau^2 = 0.3054$, $p < 0.01$

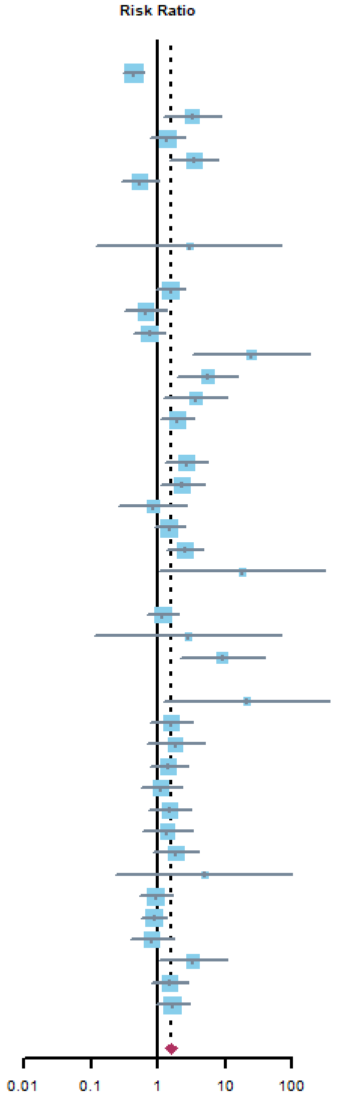

Figure S5. Forest plot of all grade ALT elevation for PD-1/PD-L1 inhibitors compared with control groups.

Study	treatment Events	treatment Total	control Events	control Total	RR	95%-CI	Weight
L.Paz-Ares.1	0	475	0	234			0.0%
Paolo A Ascierto.2	32	452	75	453	0.43	[0.29; 0.63]	4.6%
Hossein Borghaei.4.1	0	131	0	129			0.0%
Hossein Borghaei.4.2	0	268	0	268			0.0%
T.Andre.5	24	153	12	153	2.00	[1.04; 3.85]	3.8%
Joaquim Bellmunt.7	20	390	5	397	4.07	[1.54; 10.74]	2.9%
Barbara Burtness.10	17	300	25	287	0.65	[0.36; 1.18]	4.0%
Ezra EW Cohen.12	10	246	13	234	0.73	[0.33; 1.64]	3.3%
Robert L.Ferris.13	0	236	0	111			0.0%
Y.Fradet.14	1	266	0	255	2.88	[0.12; 70.28]	0.5%
Ken Kato.16	0	210	0	210			0.0%
Alexander M.M.Eggermont.17	3	502	0	502	7.00	[0.36; 135.17]	0.6%
R.L,Ferris.18	17	237	14	240	1.23	[0.62; 2.44]	3.7%
Richard S.Finn.19	63	279	22	55	0.56	[0.38; 0.83]	4.6%
Matthe D Galsky.21	12	55	4	134	7.31	[2.46; 21.68]	2.6%
Roy S.Herbst.22.1	1	339	0	309	2.73	[0.11; 66.89]	0.5%
Roy S.Herbst.22.2	0	343	0	309			0.0%
Roy S.Herbst.23	30	285	11	285	2.73	[1.39; 5.34]	3.7%
Jing Huang.24	0	228	0	228			0.0%
R.J.Kelly.25	51	532	14	260	1.78	[1.00; 3.16]	4.0%
Takashi Kojima.27	26	314	14	296	1.75	[0.93; 3.29]	3.9%
John Kuruvilla.29	6	151	5	152	1.21	[0.38; 3.87]	2.4%
Shun Lu.30	54	338	10	156	2.49	[1.30; 4.76]	3.8%
Julien Mazieres.31	43	609	12	578	3.40	[1.81; 6.38]	3.9%
Julien Mazieres.31	12	142	1	135	11.41	[1.50; 86.55]	1.2%
Tony S K Mok.33	0	637	0	615			0.0%
Robert J.Motzer.34	33	410	25	397	1.28	[0.77; 2.11]	4.3%
Keunchil Park.36	1	393	0	365	2.79	[0.11; 68.19]	0.5%
T.Powles.38	0	344	0	345			0.0%
Jean-Louis Pujol.39	0	49	0	49			0.0%
David A.Reardon.40	0	184	0	184			0.0%
Martin Reck.41	14	154	7	150	1.95	[0.81; 4.69]	3.1%
Caroline Robert.42	0	206	0	205			0.0%
Caroline Robert.43.1	23	278	13	256	1.63	[0.84; 3.15]	3.8%
Caroline Robert.43.2	14	277	13	256	1.00	[0.48; 2.08]	3.5%
Ahmet Sezer.47	22	355	12	355	1.83	[0.92; 3.65]	3.7%
Kohei Shitara.48	18	254	12	244	1.44	[0.71; 2.93]	3.6%
D.R.Spigel.49	19	282	14	265	1.28	[0.65; 2.49]	3.7%
Michiel S.van der Heijden.52	1	459	0	443	2.90	[0.12; 70.89]	0.5%
Eric P Winer.54	33	312	27	292	1.14	[0.71; 1.85]	4.3%
Li-Long Wu.56	0	128	0	128			0.0%
Bang.57	17	184	14	177	1.17	[0.59; 2.30]	3.7%
Larkin.60	35	268	5	102	2.66	[1.07; 6.61]	3.0%
Hodi.61	23	313	17	311	1.34	[0.73; 2.47]	3.9%
Carbone.62	40	267	18	263	2.19	[1.29; 3.72]	4.2%
**Random effects model**		**13235**		**11772**	**1.56**	**[1.22; 2.00]**	**100.0%**

Heterogeneity: $I^2 = 71\%$, $\tau^2 = 0.3099$, $p < 0.01$

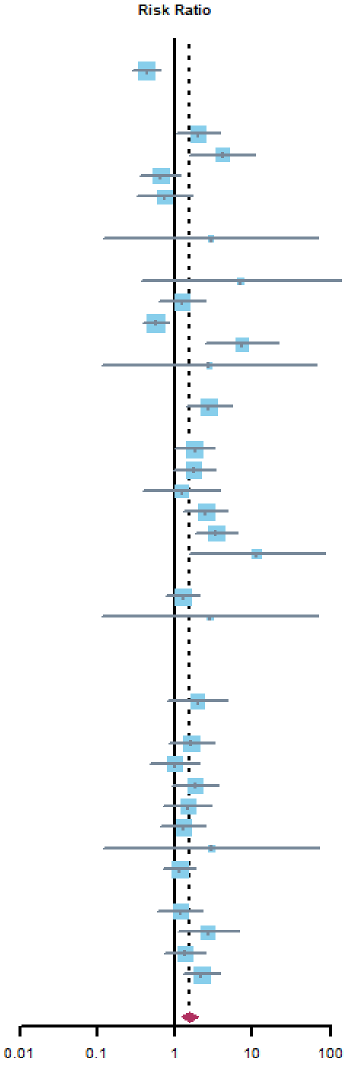

Figure S6. Forest plot of all grade AST elevation for PD-1/PD-L1 inhibitors compared with control groups.

Study	treatment Events	treatment Total	control Events	control Total	RR	95%-CI	Weight
L.Paz-Ares.1	0	475	0	234			0.0%
Paolo A Ascierto.2	1	452	1	453	1.00	[0.06; 15.97]	4.6%
Hossein Borghaei.4.1	0	131	0	129			0.0%
Hossein Borghaei.4.2	0	268	0	268			0.0%
T.Andre.5	4	153	0	153	9.00	[0.49; 165.73]	4.1%
Joaquim Bellmunt.7	2	390	0	397	5.09	[0.25; 105.67]	3.8%
Barbara Burtness.10	2	300	0	287	4.78	[0.23; 99.21]	3.8%
Ezra EW Cohen.12	1	246	0	234	2.85	[0.12; 69.71]	3.4%
Robert L.Ferris.13	0	236	0	111			0.0%
Y.Fradet.14	0	266	0	255			0.0%
Ken Kato.16	0	210	0	210			0.0%
Alexander M.M.Eggermont.17	0	502	0	502			0.0%
R.L,Ferris.18	0	237	0	240			0.0%
Richard S.Finn.19	3	279	0	134	3.37	[0.18; 64.75]	4.0%
Matthe D Galsky.21	0	55	0	55			0.0%
Roy S.Herbst.22.1	1	339	0	309	2.73	[0.11; 66.89]	3.4%
Roy S.Herbst.22.2	1	343	0	309	2.70	[0.11; 66.11]	3.4%
Roy S.Herbst.23	2	285	0	285	5.00	[0.24; 103.69]	3.8%
Jing Huang.24	0	228	0	228			0.0%
R.J.Kelly.25	1	532	0	260	1.47	[0.06; 35.90]	3.4%
Takashi Kojima.27	4	314	0	296	8.48	[0.46; 156.92]	4.1%
John Kuruvilla.29	0	151	0	152			0.0%
Shun Lu.30	0	338	0	156			0.0%
Julien Mazieres.31	2	609	0	578	4.75	[0.23; 98.64]	3.8%
Julien Mazieres.31	0	142	0	135			0.0%
Tony S K Mok.33	4	637	0	615	8.69	[0.47; 161.06]	4.1%
Robert J.Motzer.34	1	410	0	397	2.90	[0.12; 71.10]	3.4%
Keunchil Park.36	1	393	0	365	2.79	[0.11; 68.19]	3.4%
T.Powles.38	1	344	0	345	3.01	[0.12; 73.60]	3.4%
Jean-Louis Pujol.39	0	49	0	49			0.0%
David A.Reardon.40	0	184	0	184			0.0%
Martin Reck.41	0	154	0	150			0.0%
Caroline Robert.42	0	206	0	205			0.0%
Caroline Robert.43.1	4	278	0	256	8.29	[0.45; 153.20]	4.1%
Caroline Robert.43.2	1	277	0	256	2.77	[0.11; 67.76]	3.4%
Ahmet Sezer.47	2	355	0	355	5.00	[0.24; 103.78]	3.8%
Kohei Shitara.48	1	254	1	244	0.96	[0.06; 15.27]	4.6%
D.R.Spigel.49	1	282	0	265	2.82	[0.12; 68.91]	3.4%
Michiel S.van der Heijden.52	4	459	0	443	8.69	[0.47; 160.87]	4.1%
Eric P Winer.54	0	312	0	292			0.0%
Li-Long Wu.56	2	128	0	128	5.00	[0.24; 103.12]	3.8%
Bang.57	1	184	0	177	2.89	[0.12; 70.38]	3.4%
Larkin.60	0	268	0	102			0.0%
Hodi.61	2	313	1	311	1.99	[0.18; 21.80]	6.1%
Carbone.62	1	267	0	263	2.96	[0.12; 72.21]	3.4%
**Random effects model**		**13235**		**11772**	**3.54**	**[1.96; 6.38]**	**100.0%**

Heterogeneity: $I^2 = 0\%$, $\tau^2 = 0$, $p = 1.00$

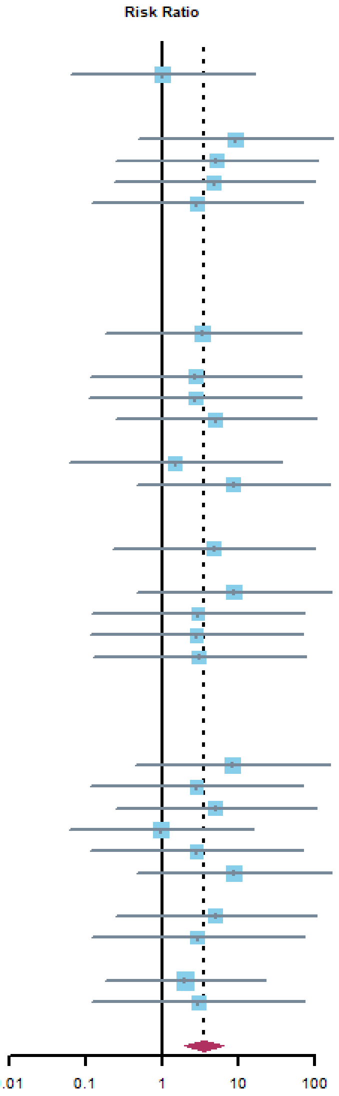

Figure S7. Forest plot of all grade hepatitis for PD-1/PD-L1 inhibitors compared with control groups.

Study	treatment Events	treatment Total	control Events	control Total	RR	95%-CI	Weight
L.Paz-Ares.1	55	475	4	234	6.77	[2.48; 18.47]	3.2%
Paolo A Ascierto.2	54	452	37	453	1.46	[0.98; 2.18]	5.0%
Hossein Borghaei.4.1	1	131	0	129	2.95	[0.12; 71.86]	0.7%
Hossein Borghaei.4.2	19	268	0	268	39.00	[2.37; 642.64]	0.8%
T.Andre.5	19	153	3	153	6.33	[1.91; 20.96]	2.7%
Joaquim Bellmunt.7	36	390	0	397	74.31	[4.58; 1206.48]	0.8%
Barbara Burtness.10	55	300	18	287	2.92	[1.76; 4.85]	4.7%
Ezra EW Cohen.12	37	246	9	234	3.91	[1.93; 7.92]	4.1%
Robert L.Ferris.13	0	236	0	111			0.0%
Y.Fradet.14	17	266	3	255	5.43	[1.61; 18.31]	2.7%
Ken Kato.16	0	210	0	210			0.0%
Alexander M.M.Eggermont.17	75	502	14	502	5.36	[3.07; 9.35]	4.6%
R.L,Ferris.18	33	237	5	240	6.68	[2.65; 16.83]	3.5%
Richard S.Finn.19	14	279	7	134	0.96	[0.40; 2.32]	3.6%
Matthe D Galsky.21	5	55	2	55	2.50	[0.51; 12.34]	2.0%
Roy S.Herbst.22.1	28	339	1	309	25.52	[3.49; 186.46]	1.5%
Roy S.Herbst.22.2	28	343	1	309	25.22	[3.45; 184.30]	1.5%
Roy S.Herbst.23	25	285	3	285	8.33	[2.54; 27.29]	2.8%
Jing Huang.24	38	228	3	228	12.67	[3.97; 40.45]	2.8%
R.J.Kelly.25	65	532	5	260	6.35	[2.59; 15.59]	3.5%
Takashi Kojima.27	36	314	7	296	4.85	[2.19; 10.72]	3.8%
John Kuruvilla.29	23	151	2	152	11.58	[2.78; 48.24]	2.3%
Shun Lu.30	0	338	0	156			0.0%
Julien Mazieres.31	0	609	0	578			0.0%
Julien Mazieres.31	13	142	0	135	25.67	[1.54; 427.66]	0.8%
Tony S K Mok.33	77	637	9	615	8.26	[4.18; 16.33]	4.2%
Robert J.Motzer.34	28	410	6	397	4.52	[1.89; 10.79]	3.6%
Keunchil Park.36	24	393	1	365	22.29	[3.03; 163.93]	1.4%
T.Powles.38	41	344	2	345	20.56	[5.01; 84.33]	2.3%
Jean-Louis Pujol.39	2	49	0	49	5.00	[0.25; 101.51]	0.7%
David A.Reardon.40	0	184	0	184			0.0%
Martin Reck.41	14	154	2	150	6.82	[1.58; 29.49]	2.2%
Caroline Robert.42	13	206	2	205	6.47	[1.48; 28.30]	2.2%
Caroline Robert.43.1	30	278	5	256	5.53	[2.18; 14.02]	3.4%
Caroline Robert.43.2	25	277	5	256	4.62	[1.80; 11.89]	3.4%
Ahmet Sezer.47	0	355	0	355			0.0%
Kohei Shitara.48	21	254	10	244	2.02	[0.97; 4.20]	4.0%
D.R.Spigel.49	16	282	0	265	31.01	[1.87; 514.37]	0.8%
Michiel S.van der Heijden.52	2	459	0	443	4.83	[0.23; 100.24]	0.7%
Eric P Winer.54	24	312	4	292	5.62	[1.97; 15.99]	3.1%
Li-Long Wu.56	15	128	0	128	31.00	[1.87; 512.61]	0.8%
Bang.57	1	184	0	177	2.89	[0.12; 70.38]	0.7%
Larkin.60	26	268	0	102	20.23	[1.24; 328.94]	0.8%
Hodi.61	34	313	16	311	2.11	[1.19; 3.75]	4.5%
Carbone.62	20	267	7	263	2.81	[1.21; 6.54]	3.7%
**Random effects model**		**13235**		**11772**	**5.29**	**[4.00; 6.99]**	**100.0%**

Heterogeneity: $I^2 = 59\%$, $\tau^2 = 0.3630$, $p < 0.01$

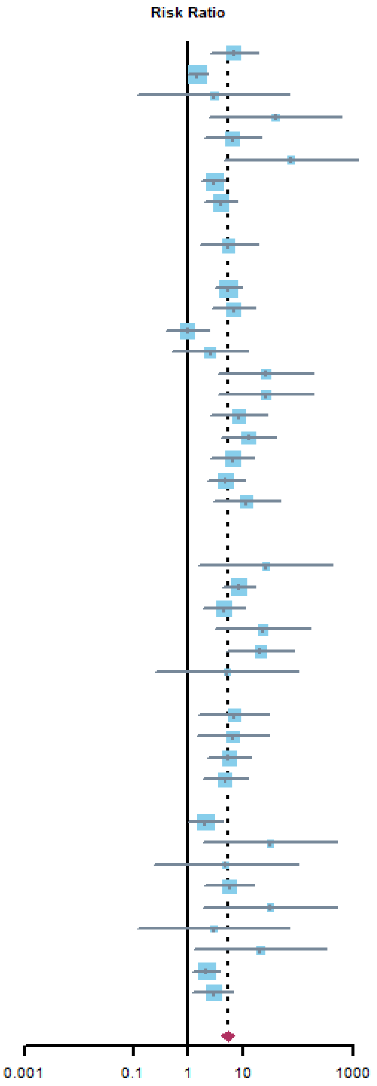

Figure S8. Forest plot of all grade hypothyroid for PD-1/PD-L1 inhibitors compared with control groups.

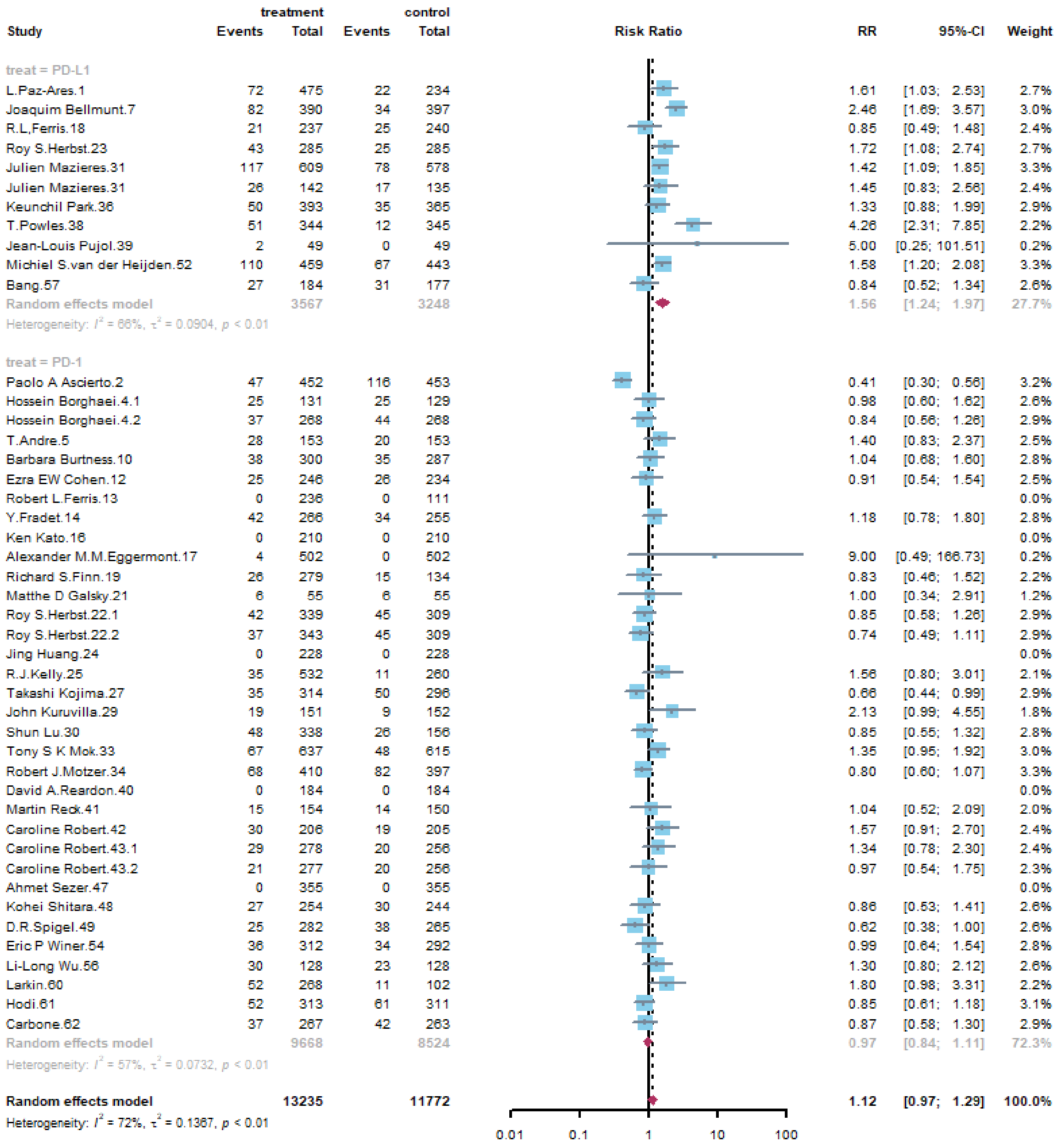

Figure S9. Forest plot of all grade fever for PD-1 or PD-L1 inhibitors compared with control groups.

Study	treatment Events	treatment Total	control Events	control Total	RR	95%-CI	Weight
treat = PD-L1							
L.Paz-Ares.1	52	475	21	234	1.22	[0.75; 1.98]	3.2%
Joaquim Bellmunt.7	36	390	8	397	4.58	[2.16; 9.73]	1.8%
R.L,Ferris.18	13	237	16	240	0.82	[0.40; 1.67]	1.9%
Roy S.Herbst.23	31	285	18	285	1.72	[0.99; 3.01]	2.7%
Julien Mazieres.31	62	609	46	578	1.28	[0.89; 1.84]	4.1%
Julien Mazieres.31	16	142	10	135	1.52	[0.72; 3.23]	1.8%
Keunchil Park.36	30	393	19	365	1.47	[0.84; 2.56]	2.7%
T.Powles.38	24	344	9	345	2.67	[1.26; 5.67]	1.8%
Jean-Louis Pujol.39	0	49	0	49			0.0%
Michiel S.van der Heijden.52	38	459	27	443	1.36	[0.84; 2.19]	3.2%
Bang.57	0	184	0	177			0.0%
Random effects model		3567		3248	1.55	[1.19; 2.01]	23.0%
Heterogeneity: $I^2 = 48\%$, $\tau^2 = 0.0740$, $p = 0.05$							
treat = PD-1							
Paolo A Ascierto.2	109	452	148	453	0.74	[0.60; 0.91]	5.6%
Hossein Borghaei.4.1	18	131	9	129	1.97	[0.92; 4.22]	1.7%
Hossein Borghaei.4.2	32	268	32	268	1.00	[0.63; 1.58]	3.3%
T.Andre.5	21	153	22	153	0.95	[0.55; 1.66]	2.7%
Barbara Burtness.10	36	300	24	287	1.43	[0.88; 2.34]	3.1%
Ezra EW Cohen.12	21	246	22	234	0.91	[0.51; 1.61]	2.6%
Robert L.Ferris.13	0	236	0	111			0.0%
Y.Fradet.14	13	266	13	255	0.96	[0.45; 2.03]	1.8%
Ken Kato.16	0	210	0	210			0.0%
Alexander M.M.Eggermont.17	96	502	93	502	1.03	[0.80; 1.33]	5.2%
Richard S.Finn.19	19	279	5	134	1.83	[0.70; 4.78]	1.2%
Matthe D Galsky.21	6	55	5	55	1.20	[0.39; 3.70]	0.9%
Roy S.Herbst.22.1	36	339	19	309	1.73	[1.01; 2.95]	2.8%
Roy S.Herbst.22.2	28	343	19	309	1.33	[0.76; 2.33]	2.6%
Jing Huang.24	0	228	0	228			0.0%
R.J.Kelly.25	42	532	31	260	0.66	[0.43; 1.03]	3.5%
Takashi Kojima.27	1	314	0	296	2.83	[0.12; 69.15]	0.1%
John Kuruvilla.29	0	151	0	152			0.0%
Shun Lu.30	19	338	3	156	2.92	[0.88; 9.73]	0.8%
Tony S K Mok.33	44	637	51	615	0.83	[0.57; 1.23]	3.9%
Robert J.Motzer.34	57	410	55	397	1.00	[0.71; 1.42]	4.3%
David A.Reardon.40	8	184	8	184	1.00	[0.38; 2.61]	1.2%
Martin Reck.41	9	154	7	150	1.25	[0.48; 3.28]	1.2%
Caroline Robert.42	31	206	28	205	1.10	[0.69; 1.77]	3.2%
Caroline Robert.43.1	45	278	27	256	1.53	[0.98; 2.40]	3.4%
Caroline Robert.43.2	33	277	27	256	1.13	[0.70; 1.82]	3.2%
Ahmet Sezer.47	0	355	0	355			0.0%
Kohei Shitara.48	16	254	16	244	0.96	[0.49; 1.88]	2.1%
D.R.Spigel.49	27	282	17	265	1.49	[0.83; 2.67]	2.5%
Eric P Winer.54	44	312	33	292	1.25	[0.82; 1.90]	3.6%
Li-Long Wu.56	0	128	0	128			0.0%
Larkin.60	41	268	12	102	1.30	[0.71; 2.37]	2.4%
Hodi.61	69	313	75	311	0.91	[0.69; 1.22]	4.9%
Carbone.62	25	267	31	263	0.79	[0.48; 1.31]	3.0%
Random effects model		9668		8524	1.06	[0.95; 1.18]	77.0%
Heterogeneity: $I^2 = 26\%$, $\tau^2 = 0.0203$, $p = 0.10$							
**Random effects model**		**13235**		**11772**	**1.17**	**[1.04; 1.32]**	**100.0%**
Heterogeneity: $I^2 = 45\%$, $\tau^2 = 0.0505$, $p < 0.01$							

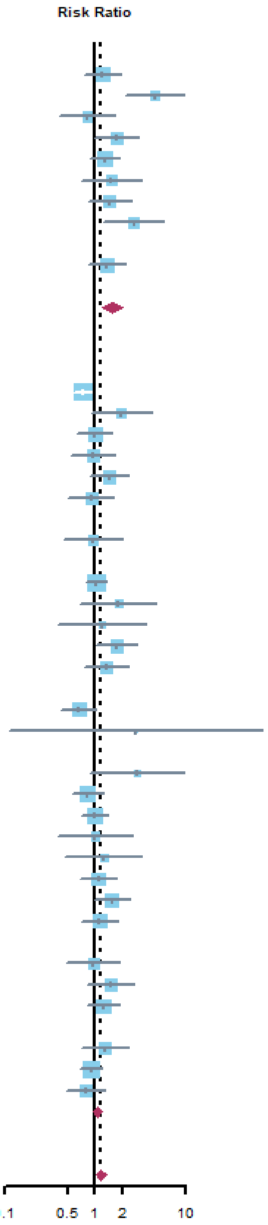

Figure S10. Forest plot of all grade headache for PD-1 or PD-L1 inhibitors compared with control groups.

Study	treatment Events	treatment Total	control Events	control Total	RR	95%-CI	Weight
**frequency = q2w**							
L.Paz-Ares.1	38	475	10	234	1.87	[0.95; 3.69]	3.5%
Paolo A Ascierto.2	63	452	36	453	1.75	[1.19; 2.59]	4.0%
Robert L.Ferris.13	0	236	0	111			0.0%
Ken Kato.16	0	210	0	210			0.0%
R.L.Ferris.18	0	237	0	240			0.0%
Jing Huang.24	0	228	0	228			0.0%
R.J.Kelly.25	38	532	13	260	1.43	[0.77; 2.63]	3.6%
Shun Lu.30	11	338	10	156	0.51	[0.22; 1.17]	3.2%
Robert J.Motzer.34	40	410	14	397	2.77	[1.53; 5.00]	3.7%
Keunchil Park.36	19	393	43	365	0.41	[0.24; 0.69]	3.8%
T.Powles.38	30	344	10	345	3.01	[1.49; 6.06]	3.4%
David A.Reardon.40	0	184	0	184			0.0%
Caroline Robert.42	13	206	8	205	1.62	[0.68; 3.82]	3.1%
Caroline Robert.43.1	33	278	8	256	3.80	[1.79; 8.07]	3.3%
D.R.Spigel.49	0	282	0	265			0.0%
Bang.57	0	184	0	177			0.0%
Larkin.60	26	268	10	102	0.99	[0.49; 1.98]	3.5%
Hodi.61	31	313	22	311	1.40	[0.83; 2.36]	3.8%
Carbone.62	26	267	15	263	1.71	[0.93; 3.15]	3.6%
Random effects model		5837		4762	1.48	[1.03; 2.15]	42.5%
Heterogeneity: $I^2 = 76\%$, $\tau^2 = 0.3137$, $p < 0.01$							
**frequency = q3w**							
Hossein Borghaei.4.1	3	131	15	129	0.20	[0.06; 0.66]	2.4%
Hossein Borghaei.4.2	18	268	35	268	0.51	[0.30; 0.88]	3.8%
T.Andre.5	1	153	0	153	3.00	[0.12; 73.07]	0.7%
Joaquim Bellmunt.7	21	390	6	397	3.56	[1.45; 8.73]	3.0%
Barbara Burtness.10	0	300	0	287			0.0%
Ezra EW Cohen.12	0	246	0	234			0.0%
Y.Fradet.14	14	266	17	255	0.79	[0.40; 1.57]	3.5%
Alexander M.M.Eggermont.17	36	502	25	502	1.44	[0.88; 2.36]	3.9%
Richard S.Finn.19	14	279	5	134	1.34	[0.49; 3.66]	2.8%
Matthe D Galsky.21	5	55	1	55	5.00	[0.60; 41.42]	1.2%
Roy S.Herbst.22.1	22	339	34	309	0.59	[0.35; 0.99]	3.8%
Roy S.Herbst.22.2	15	343	34	309	0.40	[0.22; 0.72]	3.7%
Roy S.Herbst.23	0	285	0	285			0.0%
Takashi Kojima.27	8	314	25	296	0.30	[0.14; 0.66]	3.3%
John Kuruvilla.29	0	151	0	152			0.0%
Julien Mazieres.31	44	609	91	578	0.46	[0.33; 0.65]	4.1%
Julien Mazieres.31	10	142	18	135	0.53	[0.25; 1.10]	3.4%
Tony S K Mok.33	32	637	70	615	0.44	[0.29; 0.66]	4.0%
Jean-Louis Pujol.39	0	49	0	49			0.0%
Martin Reck.41	10	154	8	150	1.22	[0.49; 3.00]	3.0%
Caroline Robert.43.2	15	277	8	256	1.73	[0.75; 4.02]	3.1%
Ahmet Sezer.47	0	355	0	355			0.0%
Kohei Shitara.48	3	254	0	244	6.72	[0.35; 129.52]	0.7%
Michiel S.van der Heijden.52	26	459	56	443	0.45	[0.29; 0.70]	3.9%
Eric P Winer.54	1	312	0	292	2.81	[0.11; 68.66]	0.7%
Li-Long Wu.56	4	128	9	128	0.44	[0.14; 1.41]	2.5%
Random effects model		7398		7010	0.71	[0.53; 0.97]	57.5%
Heterogeneity: $I^2 = 69\%$, $\tau^2 = 0.2671$, $p < 0.01$							
**Random effects model**		**13235**		**11772**	**1.00**	**[0.75; 1.32]**	**100.0%**
Heterogeneity: $I^2 = 81\%$, $\tau^2 = 0.4636$, $p < 0.01$							

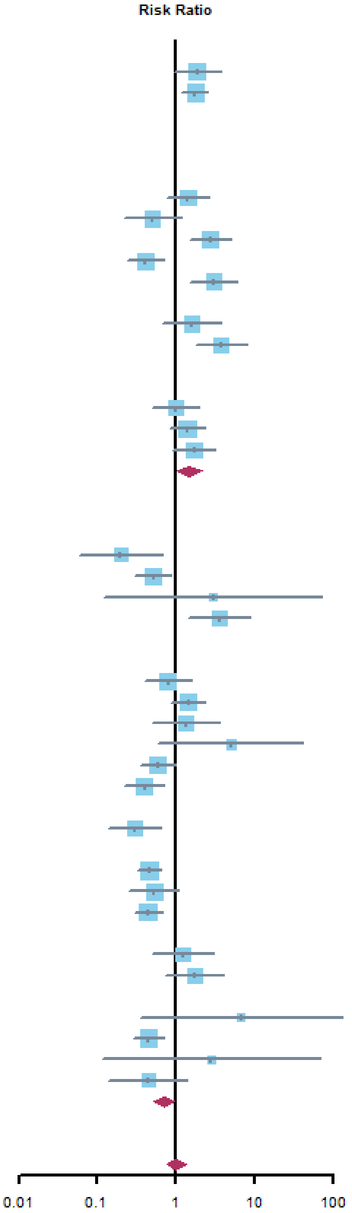

Figure S11. Forest plot of myalgia in subgroup analysis by different treatment frequency

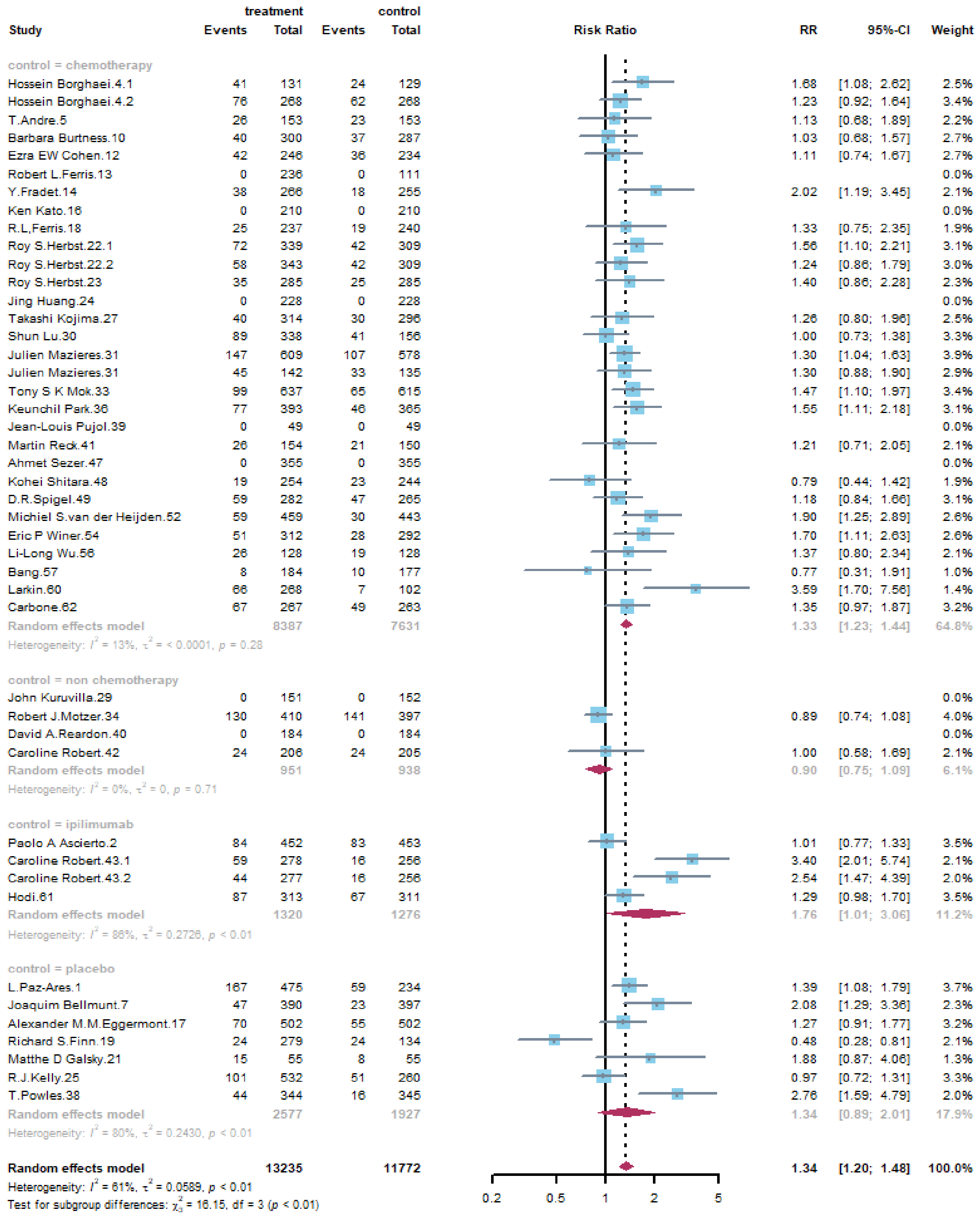

Figure S12. Forest plot of all grade cough in subgroup analysis by different control groups.

Study	treatment Events	treatment Total	control Events	control Total	RR	95%-CI	Weight
**control = chemotherapy**							
Hossein Borghaei.4.1	48	131	40	129	1.18	[0.84; 1.66]	3.5%
Hossein Borghaei.4.2	69	268	66	268	1.05	[0.78; 1.40]	4.1%
T.Andre.5	21	153	15	153	1.40	[0.75; 2.61]	1.5%
Barbara Burtness.10	42	300	20	287	2.01	[1.21; 3.34]	2.1%
Ezra EW Cohen.12	34	246	27	234	1.20	[0.75; 1.92]	2.3%
Robert L.Ferris.13	0	236	0	111			0.0%
Y.Fradet.14	33	266	24	255	1.32	[0.80; 2.17]	2.2%
Ken Kato.16	0	210	0	210			0.0%
R.L,Ferris.18	30	237	19	240	1.60	[0.93; 2.76]	1.9%
Roy S.Herbst.22.1	85	339	58	309	1.34	[0.99; 1.80]	4.1%
Roy S.Herbst.22.2	73	343	58	309	1.13	[0.83; 1.54]	3.9%
Roy S.Herbst.23	41	285	27	285	1.52	[0.96; 2.40]	2.5%
Jing Huang.24	0	228	0	228			0.0%
Takashi Kojima.27	31	314	17	296	1.72	[0.97; 3.04]	1.8%
Shun Lu.30	51	338	24	156	0.98	[0.63; 1.53]	2.5%
Julien Mazieres.31	130	609	115	578	1.07	[0.86; 1.34]	5.2%
Julien Mazieres.31	43	142	28	135	1.46	[0.97; 2.21]	2.8%
Tony S K Mok.33	107	637	70	615	1.48	[1.12; 1.95]	4.3%
Keunchil Park.36	75	393	54	365	1.29	[0.94; 1.78]	3.8%
Jean-Louis Pujol.39	0	49	0	49			0.0%
Martin Reck.41	34	154	24	150	1.38	[0.86; 2.21]	2.3%
Ahmet Sezer.47	6	355	7	355	0.86	[0.29; 2.53]	0.6%
Kohei Shitara.48	12	254	10	244	1.15	[0.51; 2.62]	1.0%
D.R.Spigel.49	63	282	45	265	1.32	[0.93; 1.86]	3.5%
Michiel S.van der Heijden.52	69	459	46	443	1.45	[1.02; 2.05]	3.4%
Eric P Winer.54	39	312	30	292	1.22	[0.78; 1.91]	2.5%
Li-Long Wu.56	15	128	16	128	0.94	[0.48; 1.81]	1.4%
Bang.57	9	184	19	177	0.46	[0.21; 0.98]	1.1%
Larkin.60	53	268	17	102	1.19	[0.72; 1.95]	2.2%
Carbone.62	67	267	43	263	1.53	[1.09; 2.16]	3.5%
Random effects model		8387		7631	1.26	[1.17; 1.37]	70.2%
Heterogeneity: $I^2 = 0\%$, $\tau^2 = < 0.0001$, $p = 0.55$							
**control = non chemotherapy**							
John Kuruvilla.29	0	151	0	152			0.0%
Robert J.Motzer.34	97	410	109	397	0.86	[0.68; 1.09]	5.0%
David A.Reardon.40	0	184	0	184			0.0%
Caroline Robert.42	17	206	23	205	0.74	[0.41; 1.34]	1.7%
Random effects model		951		938	0.84	[0.68; 1.05]	6.6%
Heterogeneity: $I^2 = 0\%$, $\tau^2 = 0$, $p = 0.63$							
**control = ipilimumab**							
Paolo A Ascierto.2	36	452	46	453	0.78	[0.52; 1.19]	2.8%
Caroline Robert.43.1	30	278	15	256	1.84	[1.01; 3.34]	1.7%
Caroline Robert.43.2	28	277	15	256	1.73	[0.94; 3.15]	1.6%
Hodi.61	47	313	43	311	1.09	[0.74; 1.59]	3.1%
Random effects model		1320		1276	1.22	[0.83; 1.80]	9.2%
Heterogeneity: $I^2 = 60\%$, $\tau^2 = 0.0957$, $p = 0.06$							
**control = placebo**							
L.Paz-Ares.1	107	475	56	234	0.94	[0.71; 1.25]	4.3%
Joaquim Bellmunt.7	27	390	8	397	3.44	[1.58; 7.47]	1.1%
Alexander M.M.Eggermont.17	46	502	25	502	1.84	[1.15; 2.95]	2.4%
Richard S.Finn.19	18	279	15	134	0.58	[0.30; 1.11]	1.4%
Matthe D Galsky.21	15	55	7	55	2.14	[0.95; 4.84]	1.0%
R.J.Kelly.25	57	532	28	260	0.99	[0.65; 1.53]	2.7%
T.Powles.38	23	344	11	345	2.10	[1.04; 4.23]	1.3%
Random effects model		2577		1927	1.41	[0.91; 2.16]	14.1%
Heterogeneity: $I^2 = 74\%$, $\tau^2 = 0.2459$, $p < 0.01$							
**Random effects model**		**13235**		**11772**	**1.23**	**[1.13; 1.34]**	**100.0%**
Heterogeneity: $I^2 = 42\%$, $\tau^2 = 0.0245$, $p < 0.01$							
Test for subgroup differences: $\chi^2_3 = 12.03$, df = 3 ($p < 0.01$)							

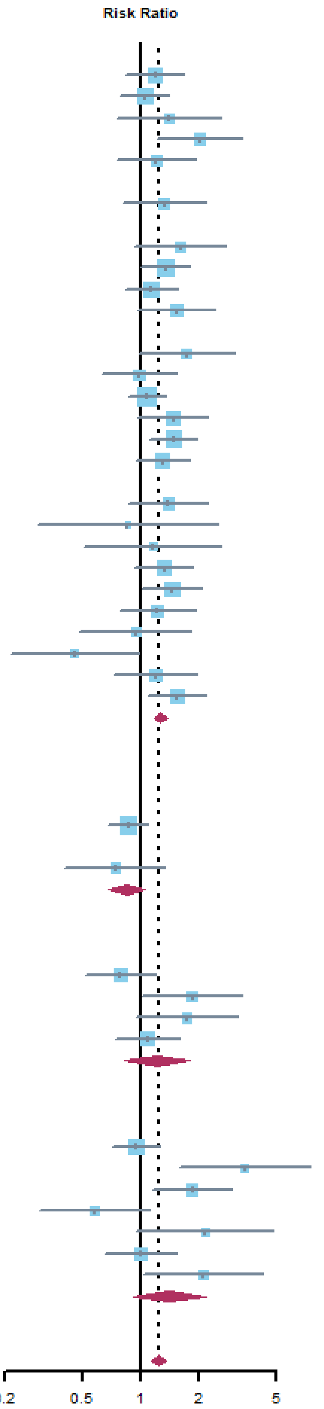

Figure S13. Forest plot of all grade dyspnea in subgroup analysis by different control groups.
