# Supplementary figures and images for: Treatment-Related Adverse Events with PD-1 or PD-L1 Inhibitors: A Systematic Review and Meta-Analysis
Source: Life (Basel). 2021 Nov 22;11(11):1277. doi: 10.3390/life11111277 (PMC8618590; doi:10.3390/life11111277)

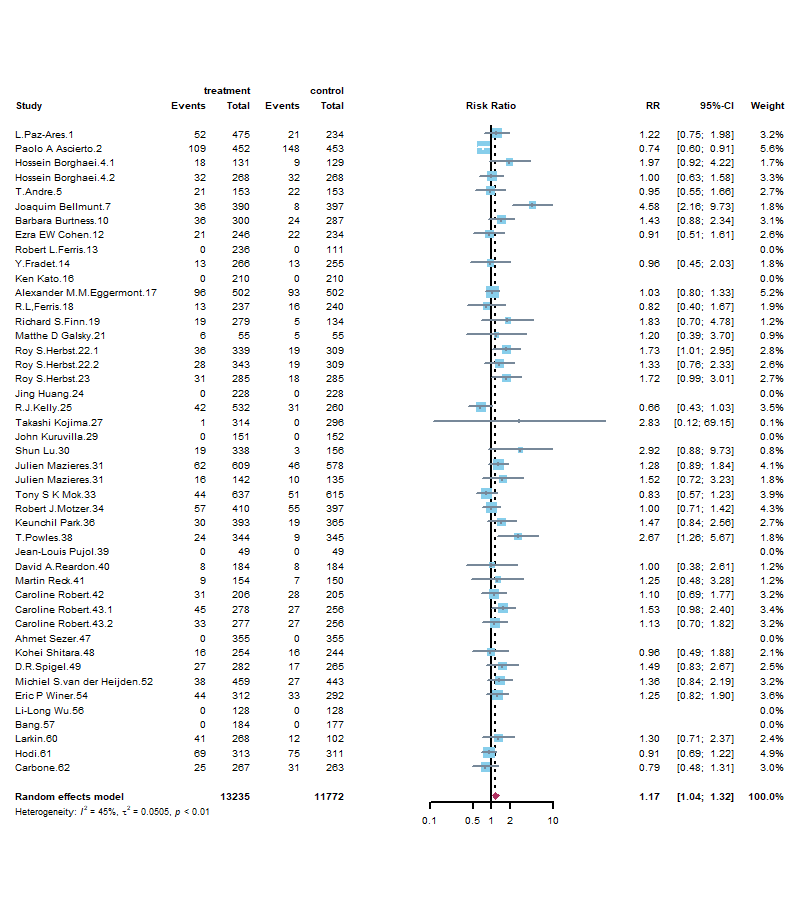

Supplement: Supplementary file 1 [file life-11-01277-s001.zip › Proofreading -- Supplimentary Files/figures supplementary/Figure S10_headache.png]

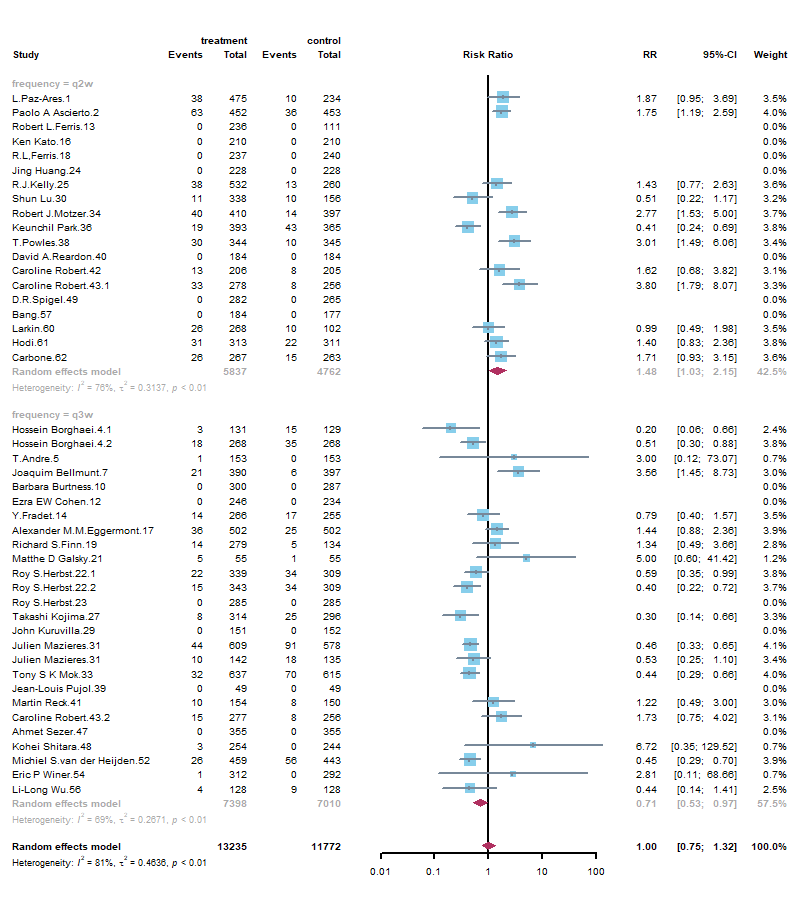

Supplement: Supplementary file 1 [file life-11-01277-s001.zip › Proofreading -- Supplimentary Files/figures supplementary/Figure S11_myalgia_frequency.png]

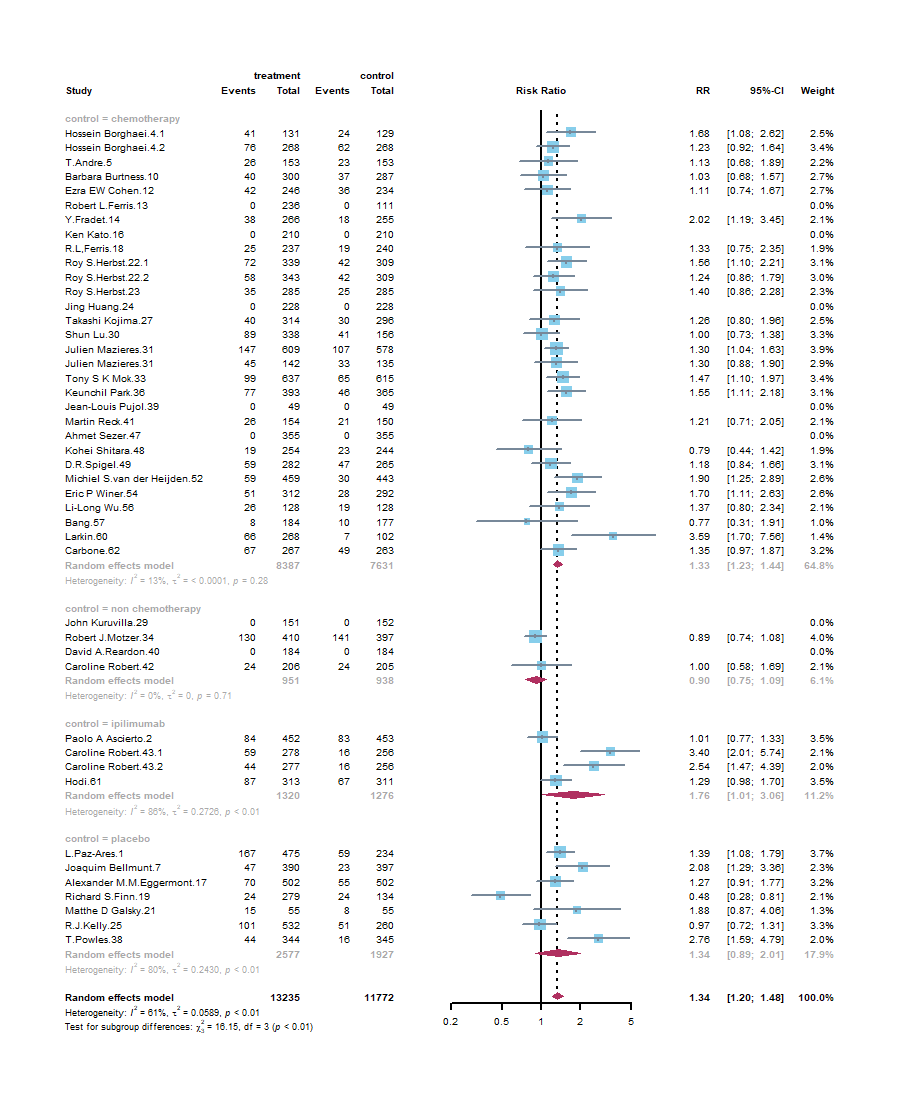

Supplement: Supplementary file 1 [file life-11-01277-s001.zip › Proofreading -- Supplimentary Files/figures supplementary/Figure S12_cough_control.png]

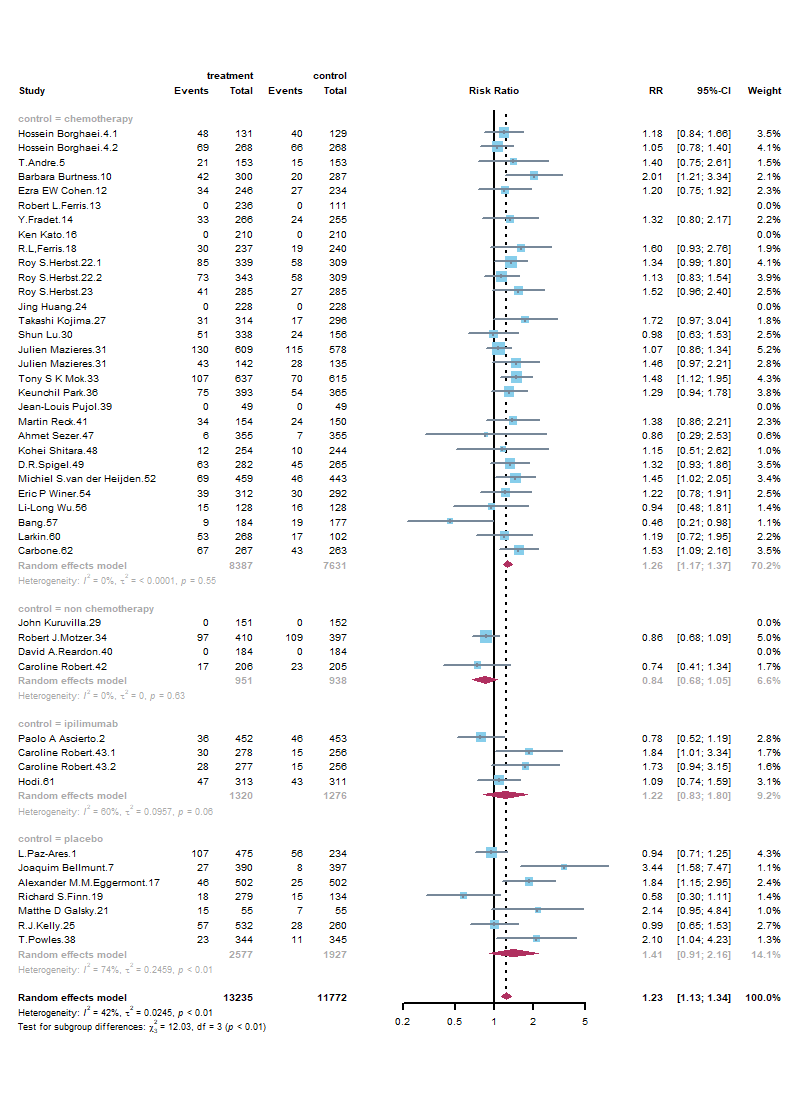

Supplement: Supplementary file 1 [file life-11-01277-s001.zip › Proofreading -- Supplimentary Files/figures supplementary/Figure S13_dyspnea_control.png]

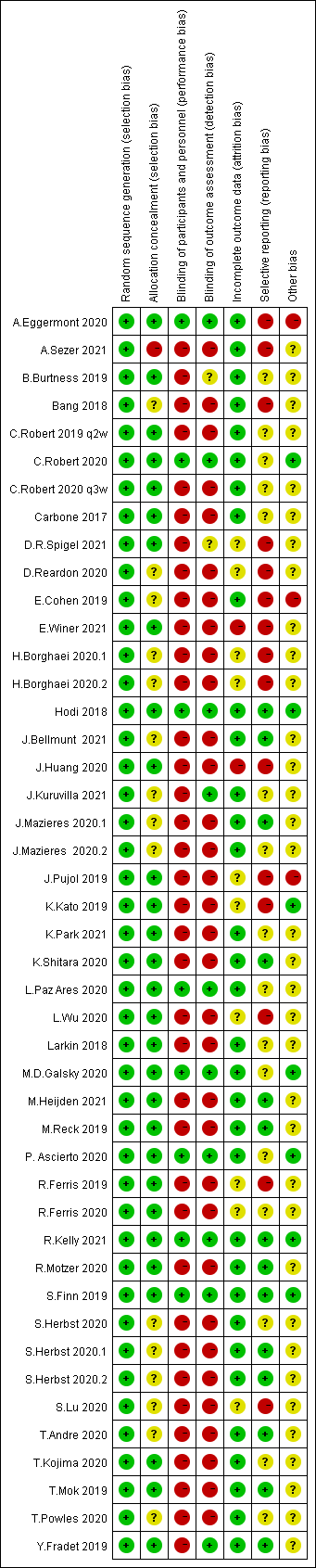

Supplement: Supplementary file 1 [file life-11-01277-s001.zip › Proofreading -- Supplimentary Files/figures supplementary/Figure S1_Risk of bias summary.png]

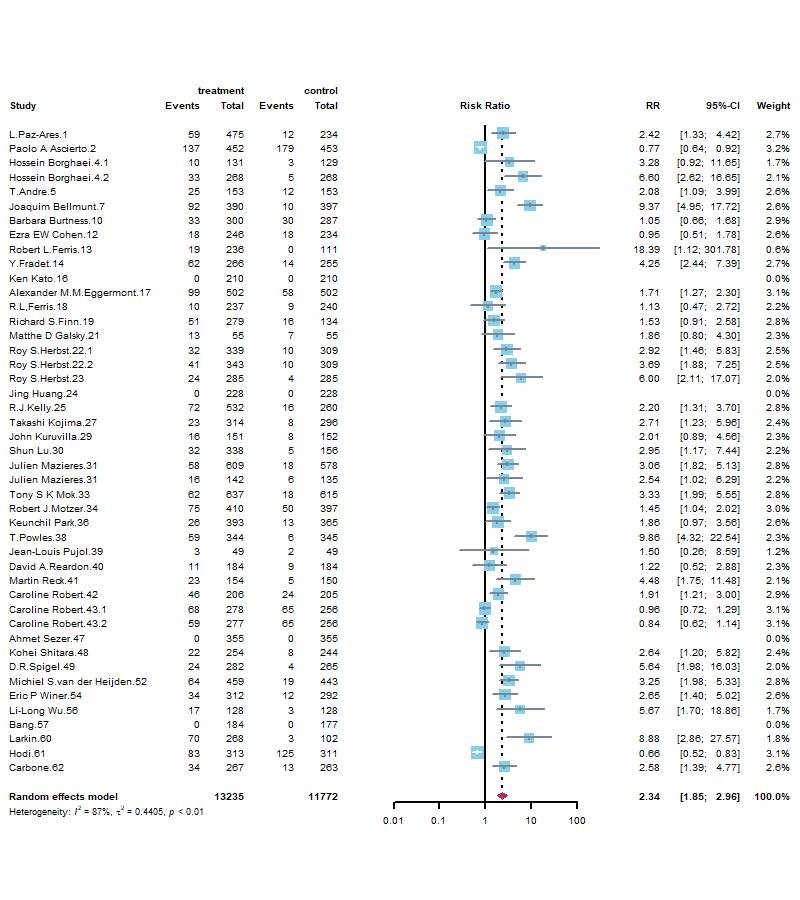

Supplement: Supplementary file 1 [file life-11-01277-s001.zip › Proofreading -- Supplimentary Files/figures supplementary/Figure S2_pruritus.png]

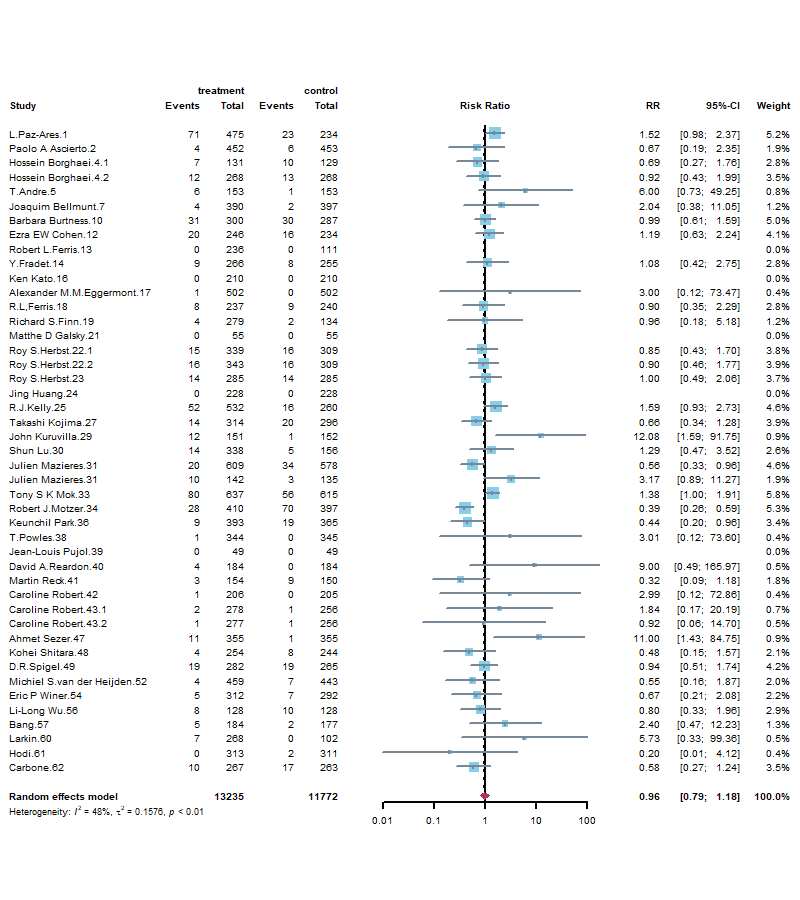

Supplement: Supplementary file 1 [file life-11-01277-s001.zip › Proofreading -- Supplimentary Files/figures supplementary/Figure S3_pneumonia.png]

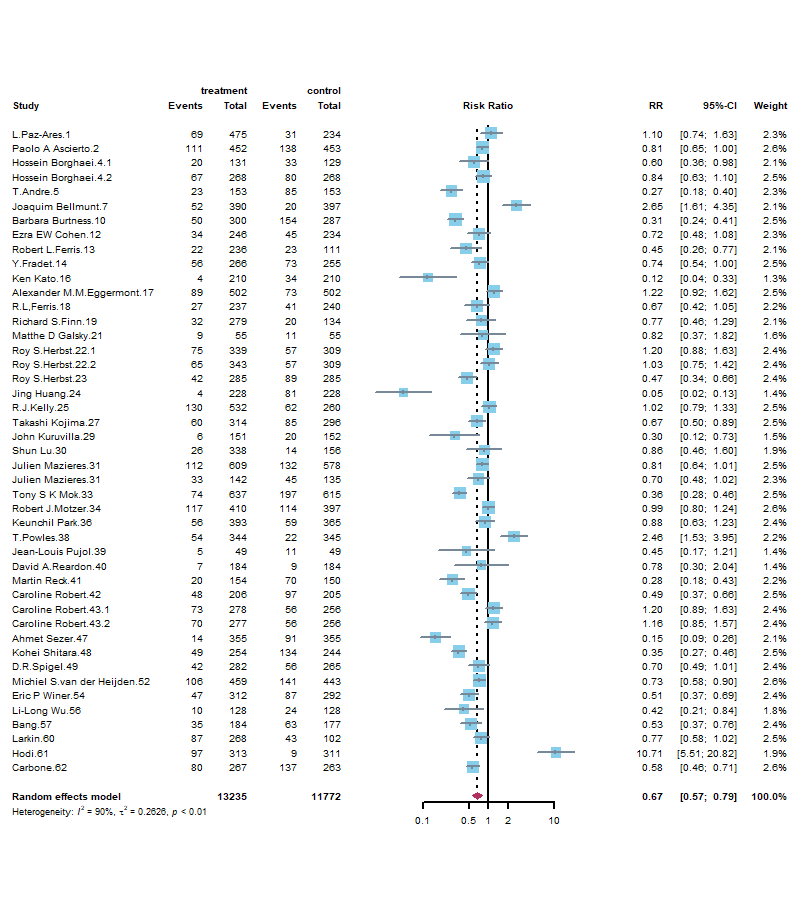

Supplement: Supplementary file 1 [file life-11-01277-s001.zip › Proofreading -- Supplimentary Files/figures supplementary/Figure S4_nausea.png]

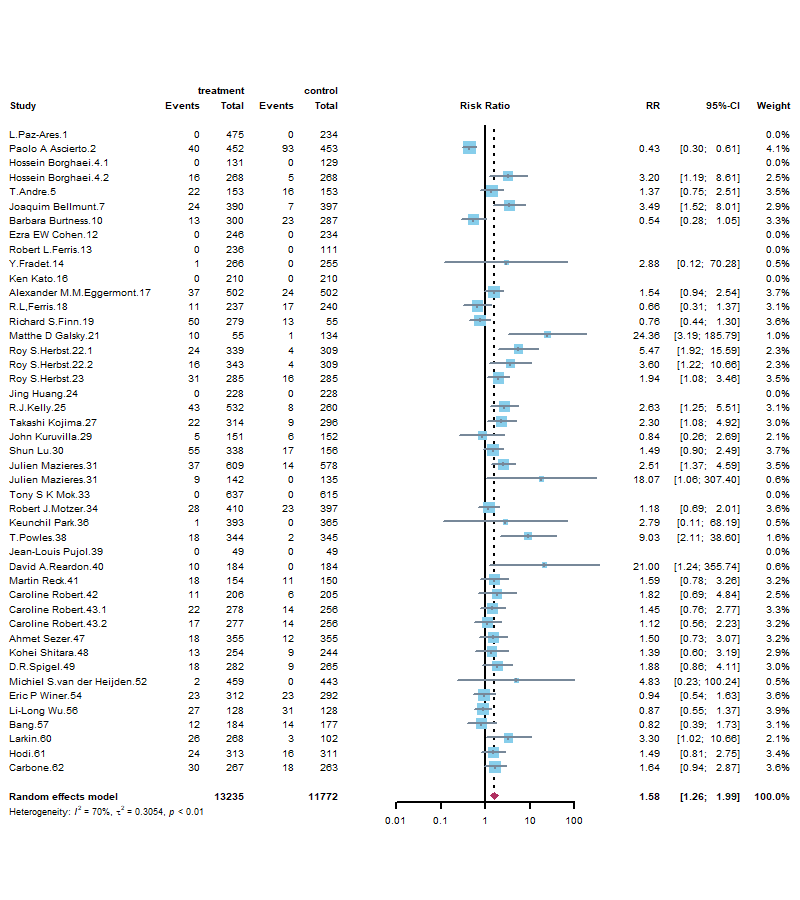

Supplement: Supplementary file 1 [file life-11-01277-s001.zip › Proofreading -- Supplimentary Files/figures supplementary/Figure S5_ALT.elevation.png]

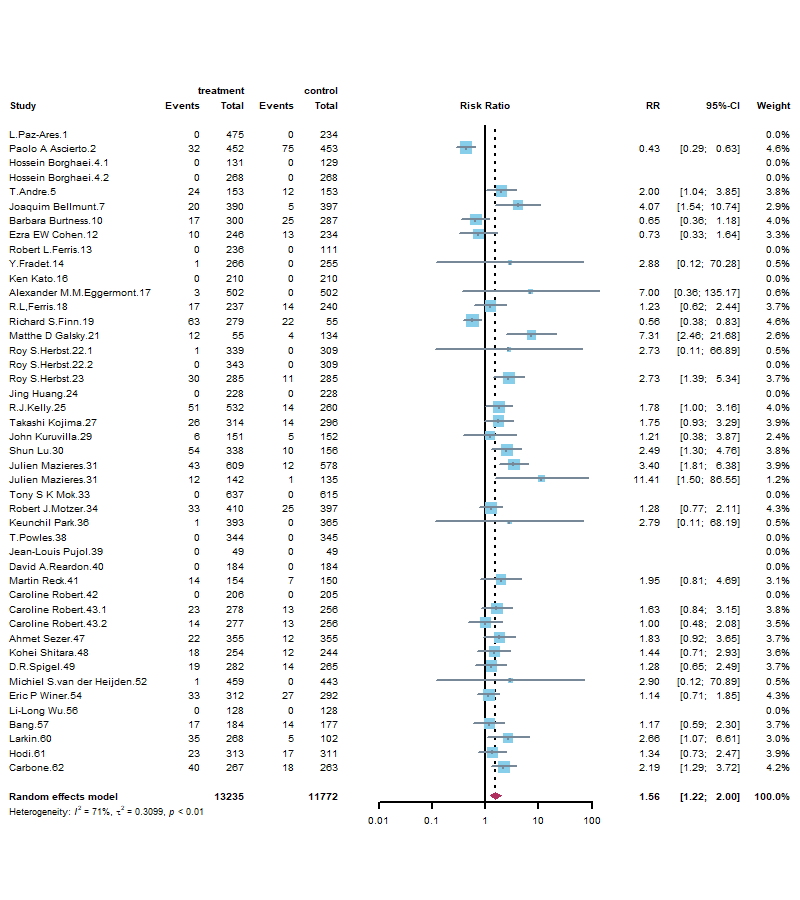

Supplement: Supplementary file 1 [file life-11-01277-s001.zip › Proofreading -- Supplimentary Files/figures supplementary/Figure S6_AST elevation.png]

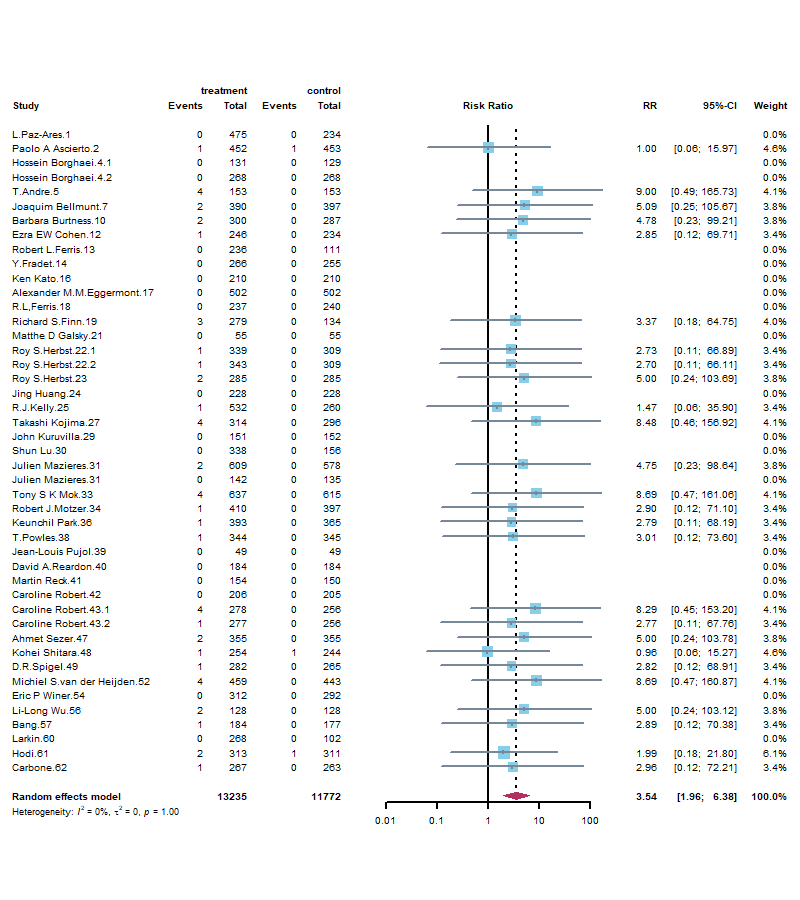

Supplement: Supplementary file 1 [file life-11-01277-s001.zip › Proofreading -- Supplimentary Files/figures supplementary/Figure S7_hepatitis.png]

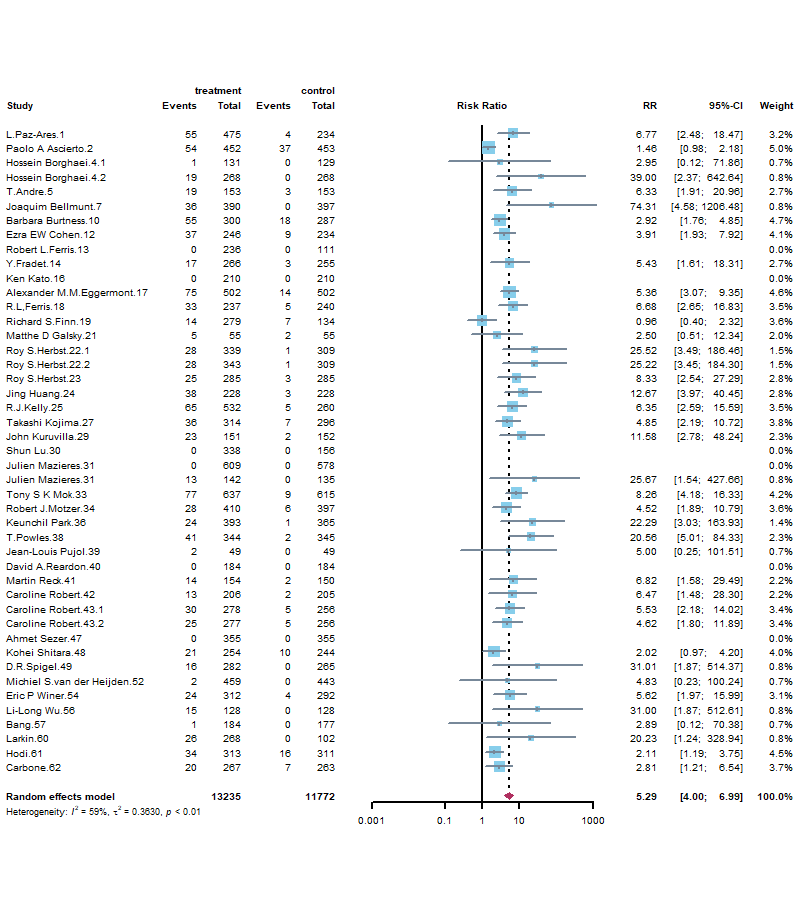

Supplement: Supplementary file 1 [file life-11-01277-s001.zip › Proofreading -- Supplimentary Files/figures supplementary/Figure S8_hypothyroid.png]

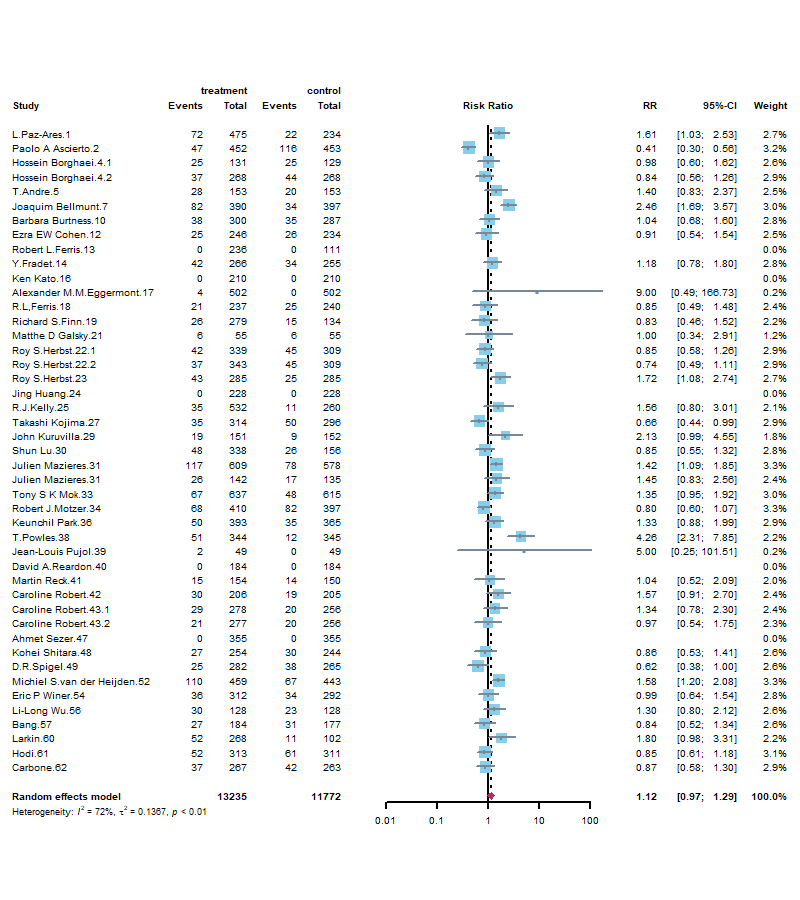

Supplement: Supplementary file 1 [file life-11-01277-s001.zip › Proofreading -- Supplimentary Files/figures supplementary/Figure S9_fever.png]
